# Radiation-assisted tailoring of swelling behavior and water retention of Na-CMC/PAAm hydrogels for enhancing *Beta Vulgaris* under drought stress

**DOI:** 10.1038/s41598-024-83832-3

**Published:** 2025-01-11

**Authors:** Mahmoud A. El-diehy, Ibrahim I. Farghal, Mohamed A. Amin, Mohamed Mohamady Ghobashy, Abdelatti I. Nowwar, H. M. Gayed

**Affiliations:** 1 Botany and Microbiology Department, Faculty of Science, Al-Azhar University, Cairo, Egypt; 2 Radiation Research of Polymer Chemistry Department, National Center for Radiation Research and Technology (NCRRT), Egyptian Atomic Energy Authority (EAEA), Cairo, Egypt

**Keywords:** Super absorbent gel, Global warming, Gamma irradiation, Rationalization of water, Soil enhancer, Planting material, Biotechnology, Physiology, Plant sciences

## Abstract

This study investigates the negative impact of climate change on water resources, specifically water for agricultural irrigation. It describes how to optimize swelling, gel properties and long-term water retention capacities of Na-CMC/PAAm hydrogels for managing drought stress of Sugar beet plants through techniques such as changing the composition, synthetic conditions and chemical modification. Gamma radiation-induced free radical copolymerization was used to synthesize superabsorbent hydrogels using sodium carboxymethyl cellulose (Na-CMC) and acrylamide (AAm). The study also explored how varying Na-CMC/AAm ratio and radiation dose influence their swelling behaviour, gel fraction, and water retention. FTIR showed that CMC and PAAm components are part of the hydrogel structure. The equilibrium swelling reached a maximum value of ~ 500 g/g at a Na-CMC/AAm ratio of 60/40. High content of AAm reduced swelling because it caused increased hydrophobicity while high radiation doses up to 50 kGy increased crosslinking resulting in improved but limited swelling from 65 to 85 (g/g). After the second cycle, KOH modification reached maximum swelling capacity by introducing anionic carboxylate groups up to 415 (g/g). SEM images revealed uniform pores in an unmodified scaffold while larger cavities were formed upon modification facilitating Water absorption. Surprisingly, the improved hydrogels retained more water: about 75% even after 16 days as opposed to a 50% drop within five days in the case of unmodified ones. This hydrogel significantly enhanced shoot length by 18%, root length by 32%, fresh weight shoot by 15%, and dry weight shoot by 15% under severe drought conditions. As a result, yield increased by 22%, proteins went up by 19%, and carbohydrates rose by 13%. Leaf chlorophyll content increased with a corresponding decline in stress enzymes indicating decreased oxidative damage. This eco-friendly Na-CMC/PAAm-based hydrogel seems to have potential use for addressing water scarcity and agricultural challenges.

## Introduction

Climate change is expected to modify precipitation patterns, with many climate models indicating a decrease in total rainfall over Egypt^1^. Drought is one of the major challenges faced by Egypt, leading the Egyptian government to look for ways through which it can address this problem. The drought has been confronted by the Egyptian government through several fronts such as research and innovation; investing in research and innovation plays a crucial role in developing new technologies and strategies to mitigate drought stress; The governments support studies that are aimed at water management, climate adaptation and sustainable agriculture that can enhance resilience when faced with changing environment.

Superabsorbent hydrogels have emerged as an encouraging class of functional materials which offer a workable solution to water scarcity in agriculture^2^. These hydrogels have a unique property of swelling to many hundreds of times their weight in water, predominantly due to the cross-linked polymeric structures^3,4^. When these hydrogels are deposited in the organo-mineral matrices of the soils, they function as water sinks releasing water to the surrounding environment and enhancing plant productivity during periods of water stress. Of the various hydrogel systems, the bacterial cellulose and its allied varieties have indeed captured the lion’s share of research interest as they are biodegradable, biomaterial deserving, and more importantly, recyclable^5^. Thus, sodium carboxymethyl cellulose (Na-CMC), an anionic non-swelling cellulose derivative soluble in water, has become the focus for developing superabsorbent hydrogels^6^. The distinctive feature in the molecular structure of carboxymethyl cellulose is the negatively charged carboxylate groups located along the cellulose backbone which contributes to the high water-absorbing capacity of the material and the ability to create hydrophilic hydrogels. Some efforts to improve further Na-CMC-based hydrogels are swelling and water uptake capabilities are reviewed as follows; Co-polymerization using synthetic monomers and Chemical modification^7^.

Concerning the application of synthetic polymers in the context of hydrogels, there is the use of PAAm or polyacrylamide, also widely used in hydrogels. PAAm is one of the most hydrophilic polymers; this is mainly due to the presence of an amide group and hydrogen bonding with water molecules as explained by Rabiee et al.^8^. Copolymer based on Na-CMC and PAAm has provided hydrogels with enhanced swelling and water retention properties that made them ideal for agricultural uses. Besides the adjustment of the hydrogel composition, the synthesis methods are the major factors affecting these materials’ structure and performance. Radiation-induced polymerization methods most often gamma irradiation have been made environment friendly and effective for hydrogel production^9,10^. Gamma rays induce free radical generation and hence promote crosslinking reactions and the development of covalent ties in between the polymer chains giving a stable and rugged three-dimensional hydrogel network^11^.

This approach avoids the use of standard chemical initiators or crosslinking agents that may harm the environment or add negative qualities to the final product. However, there is a weakness in water retention for Na-CMC/PAAm hydrogels for application in agriculture after long periods. Extended water supply is preferred to alleviate the effects of water-deficient stress on the production of crops in arid and semi-arid zones throughout the world. In response to this challenge chemical modification approaches have been proposed and tested as a way of increasing the degree of hydrophilic groups and ionic interactions within the body of the hydrogel to increase water-containing capacity. One method of modifying hydrogels is the introduction of ionic groups such as carboxylates into the structure of the hydrogel. These charged moieties can be introduced through the process of treatment with the help of alkaline solutions (KOH). Due to the ionization and formation of carboxylate groups in the hydrogel network, it becomes more hydrophilic along with increased ion-ion repulsion and ionic interaction with water molecules. Altogether, the above effects promote the enhanced WA and WR performances to make the modified hydrogels appropriate for use in arid agricultural regions.

This research work aims to.

1. Develop a new method of preparing and modulating the Na-CMC/PAAm superabsorbent hydrogels for higher water absorption and smart application in fighting drought stress in modern agricultural practices.
2. The novelty of this work lies in the unique combination of several key aspects: Thus, this paper highlighted the applicability of gamma irradiation as a green and effective method for the synthesis of Na-CMC/PAAm hydrogels without the use of chemical initiators or cross-linkers.
3. Systematic studies have to be done on how changes in the Na-CMC/PAAm compositional ratio and the radiation dose affect the swelling characteristic, gel fraction and water retention of the hydrogels. Changes of the synthesized hydrogels by the application of KOH to create charged carboxylate groups to provide more hydrophilicity and to ensure better water absorption in the long run.
4. Analysis of different characteristics of hydrogel that may include FTIR for determination of the chemical composition of the hydrogel and SEM for determination of the morphological changes and the relation between the microstructure and water retention performance.
5. Further assessment of the possibility of using the optimized, modified Na-CMC/PAAm hydrogels for agricultural drought control and future efficiency for food production in arid areas. Overall, the herein proposed integrated strategy should help advance knowledge on designing and synthesizing novel, biodegradable and efficient hydrogel systems for improving water availability and plant drought stress in agriculture to effectively feed the world and support other sustainable development goals. Finally, the substantial decrease in water usage is one of the main advantages of this work. By improving water retention and decreasing the frequency of irrigation, the SAH serves as a soil conditioner and helps preserve water resources.

## Experimental

### Materials

The sodium carboxyl methyl cellulose (Na-CMC) (with an average Mw of ~250,000 and a DS value around 0.7) was purchased from Sigma Aldrich and was used without further purification. The acrylamide monomer (99%) (AAm) and potassium hydroxide were purchased from Fluka Co. Seeds of Beta vulgaris subsp. vulgaris var. altissima were obtained at the sugar beet factory Al-Hamoul, Kafr el-Sheikh, Egypt (one of Delta’s sugar factories), and they were imported by an Egyptian agricultural organization (Schreibers Saatzuchtgesellschaft mbH), and the seeds are sold locally under the name (Romulus. The work was conducted in El-Gharbia Governorate, and sandy loam soil physical and chemical characteristics were analyzed at Ain Shams University, Faculty of Agriculture, Cairo, Egypt (Table 1). The present study complies with relevant institutional, national, and international guidelines and legislation. The researchers performed field studies using commercially available plant varieties. No appropriate permissions and/or licences for the collection of plant or seed specimens were required to conduct this research.

**Table 1:** Physiochemical estimation of the soil.

Soil texture	Sand (%) > 200–20 μm	Silt (%) 20–2 μm	Clay (%) < 2 μm
Sandy loam	64.72	21.00	14.28

CaCO3 %	Cations meq/l				Anions meq/l			ECe (dS/m)	PH at 1:2.5
4.20	K^+^	Na^+^	Mg^++^	Ca^++^	Cl^−^	HCO3^−^	CO3^−^	1.90	7.67
	0.39	8.89	3.45	5.00	8.25	4.95	*		

Conc. (mg/kg soil)						
N	K	P	Cu	Fe	Mn	Zn
34.44	217.40	15.80	5.02	17.00	0.81	4.55

### Radiation synthesis of (Na-CMC/PAAm) super absorbance hydrogel

This procedure above expounds on the preparation of Super absorbent hydrogels through copolymerization of Na-CMC and Am through gamma irradiation-induced free radical Crosslinking. The steps involved in the process are: 4cc of CMC aqueous solution 2% is prepared by dissolving 1 gm of Na-CMC in 50 cc distilled water. The solution is heated and it is stirred until a warm temperature (about 45 °C) is attained and it takes approximately one hour to dissolve all the components. Consequently, the acrylamide solution (concentration 10%) was mixed with Na-CMC solution at varied proportions of volume (v/v) (80:20, 60:40, 40:60 and 20:80). It was then stirred for 4 h, for homogeneity of the mixed cocktail. The samples that have been obtained are to be placed in sealed tubes and irradiated with gamma rays. Treatment by gamma irradiation is carried out at various radiation levels (10, 20, 30, 40, and 50 kGy) by using a Gamma Cell 220 Co60 irradiation source at the National Center for Radiation Research and Technology (NCRRT), Egyptian Atomic Energy Authority (EAEA), Cairo—Egypt. The irradiation process is done at a dose rate of approximately 0.68 kGy/h. Hydrogel samples are then irradiated and post-treatment, discs that contain the films are made by cutting the hydrogels into discs and subsequently washed in de-ionised water for 24h. This step assists in washing off any unreacted monomers or any other impurities that may be present in the polymer. The hydrogels are subsequently oven-dried and then followed by cutting the hydrogels into small portions for further study.

### Evaluation of chemical structure and morphological properties of the hydrogel

The chemical structure of the obtained (Na-CMC/PAAm) and their surface morphology with porous size were analyzed using the Fourier transform infrared spectrophotometer FTIR and scanning electron microscope (SEM), ZEISS, EVO 15, and U.K. The Fourier transform infrared spectrophotometer (ATR–FTIR) Vertex 70 FTIR spectrometer equipped with HYPERION™ series microscope (Bruker *et al*.).

### Analysis of the gel fraction (%) and swelling degree, water retention

To measure the gel fraction (%) of the obtained (Na-CMC/PAAm) hydrogel, the initial weight (*W*_*i*_) after the prepared step was measured. Then, the (Na-CMC/PAAm) hydrogel was immersed in 100 ml of distilled water and on standby for 48 h at ambient temperature. The distilled water was refreshed twice every five hours. The swollen (HEC/PAAm) hydrogel piece was placed in an oven at a temperature of 60 °C until completely dry. The dried weight was measured (*W*_*d*_), and the gel fraction (%) was calculated using Eq. (1).

1 $${\text{Gel\,\,fraction }}\left( \% \right) \, = \, \left( {{\text{W}}_{{\text{d}}} /{\text{W}}_{{\text{i}}} } \right) \, \times { 1}00$$

To calculate the swelling degree and equilibrium swelling, the dried weight of the obtained (Na-CMC/PAAm) hydrogel was measured (*W*_*d*_) and immersed in distilled water to measure the swelling rate with time (t) to give the swelled weight (*W*_*t*_).

2 $${\text{Swelling\,\,ratio}}\left( {{\text{g}}/{\text{g}}} \right) \, = \, \left( {W_{t} - W_{d} } \right)/W_{d}$$

After 48 h, the water absorption equilibrium of the (Na-CMC/PAAm) hydrogel was reached (*W*_*.E.S*_.), and the swelling equilibrium was calculated as Eq. (3).

3 $${\text{Swelling\,\,equilibrium }}\left( {{\text{g}}/{\text{g}}} \right) \, = \, \left( {W_{ES} - W_{d} } \right)/W_{d}$$

The water retention (%) was calculated by putting the weighted (Na-CMC/PAAm) hydrogel at maximum swelling in equilibrium (*W*_*.E.S*_.), the weight of hydrogel at a given time (*W*_*t*_), and the weight of the dried hydrogel (*W*_*o*_).

4 $${\text{The water retention }}\left( \% \right) \, = \, \left( {W_{t} - W_{o} } \right)/\left( {W_{o} - W_{ES} } \right) \times {1}00$$

### Chemical modification of Na-CMC/PAAm hydrogel

The specific conditions and parameters used for this alkaline hydrolysis process, as described in the previous work^12^, are critical in achieving this desired swelling and water absorbance level. The choice of alkali (in this case, KOH), concentration, temperature, and reaction time all play a role in determining the final properties of the SAH The alkaline hydrolysis of (Na-CMC/PAAm) was carried out by reaction with (10 wt%) of KOH at a temperature of 90 C for 90 min. The advantage of keeping both amino and carboxyl groups in the polymer matrix is made possible by hydrolyzing the amino group to produce the carboxylate group. By adding more hydrophilic functional groups, this dual-functional strategy improves the hydrogel’s hydrophilicity, which in turn increases its capacity to absorb water and exchange ions. This approach offers greater control over the functional groups than employing neutralized acrylic acid directly, which could result in superior mechanical stability because of increased crosslinking interactions and stronger water absorption performance.

$${\text{Polyacrylamide }} + {\text{ OH}}^{ - } \to {\text{ Polyacrylic acid }} + {\text{ NH}}_{{3}} \uparrow$$

The reaction rate is initially relatively high, and hydrolysis is rapid. This is attributed to the availability of many amide groups in the polymer chain that can readily react with the hydroxide ions. The alkaline hydrolysis of acylamino groups (–CONH_2_) catalyst by KOH is outlined as the following Equation:
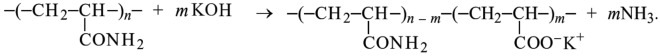


### Evaluation of the effect of superabsorbent hydrogel on the growth of Sugar beet under drought stress

Seeds of Beta vulgaris subsp. vulgaris var. altissima were obtained at the sugar beet factory Al-Hamoul, Kafr el-Sheikh, Egypt (one of Delta’s sugar factories), and they were imported by an Egyptian agricultural organization (Schreibers Saatzuchtgesellschaft mbH), and the seeds are sold locally under the name (Romulus. The work was conducted in El-Gharbia Governorate, and sandy loam soil physical and chemical characteristics were analyzed at Ain Shams University, Faculty of Agriculture, Cairo, Egypt (Table 1), the collected soil samples were air-dried and digested using the acid digestion (HNO3/H2SO4/HClO4 (5:1:1, v/v/v) for 8 h at 80 °C) method adopted by Wade et al.^13^, digestion was continued until the solution became clear, then the transparent digests were filtered using a 0.45-μm pore size cellulose nitrate membrane filter paper (Millipore) and diluted up to 50 ml with distilled water. Soluble cations, anions and HMs content were determined using a Perkin-Elmer 3100 Atomic Absorption Spectrophotometer, at the atomic spectroscopy laboratory, arid land agricultural research and service center, Faculty of Agriculture; Ain shams University, Cairo, Egypt. The phosphorus (P) and potassium (K) were determined as mentioned in water analysis. Soil reaction (pH), electrical conductivity (EC), total dissolved solids (TDS) were measured according to^14^ using pH/electric conductivity meter (914 pH/Conductometer-Metrohm AG).

For each seed, 5 g of hydrogel (Na-CMC/PAAM) had been applied for the treatment of Beta vulgaris, known as sugar beet, characterized by the detailed treatment process shown in Table 2 and Fig. 10, and they have been divided into two groups: one provided with SAH called "Hydrogel”, while the other left without being introduced to such a tolerant factor, i.e., “Control”. These treatments had passed through four different stress levels: 0%, 25%, 50% & 100% having labels, those can be read as follows: Control at zero level stress (C1); Hydrogel at zero level stress (H1); Control at level 25% (H2); Hydrogel at level 25% (H3); Control at level 50% (C3); Hydrogel at level 50 (H3); Control at maximum 100% (C4); and Hydrogal at 100% stress (H4). Make sure they can grow at different levels of stress (20 days) if the plants have no stress. 25 days, with 25% soil humidity. 30 days under a level of stress set at 50%. and finally in each 40 days for other plants that suffered a level up to a maximum of 100%). To fully understand the influence on the plant tissue, it will be sampled on two separate occasions: The first corresponded to morphological characteristics and biochemical characteristics. Morphology was described with the measured length (cm) of the shoot and root as well as the fresh and dry weight of different plant parts. The number of leaves on each plant was also counted. While biochemistry analysis included chlorophyll a and b, total chlorophyll, carotenoids, shoot phenol content^15^, shoot proline content^16^, shoot protein^17^, and shoot carbohydrate^18^, Hydrogen peroxide was measured according to Masry et al^19^, Malondialdehyde was estimated according to^20^. The levels of antioxidant enzymes (catalases, peroxidase and polyphenol oxidase), roots fresh and dry weights (gm), root length, followed by carbohydrates, proline, poly phenolic compound and protein. Chemical treatment was done on five plants for each treatment chosen.

**Table 2:** Key words of treatments.

Irrigation time (days)	Control	Hydrogel	Water stress (%)
20	C1	H1	0
25	C2	H2	25
30	C3	H3	50
40	C4	H4	100

**Statistical calculations and correlation analysis**: were done using computer programs Microsoft excel version 365 and Minitab statistical program v.19. at 0.05 level of probability^21^. The differences in the studied variables in the water samples were tested using t-test. Quantitative data with parametric distribution were done using analysis of variance the Two-way ANOVA and Post hoc-Tukey’s test. The confidence interval was set to 95% and the margin of error accepted was set to 5%.

## Results

### The chemical structure of Na-CMC/PAAm and modified hydrogels by FTIR

**Fig. 1:**
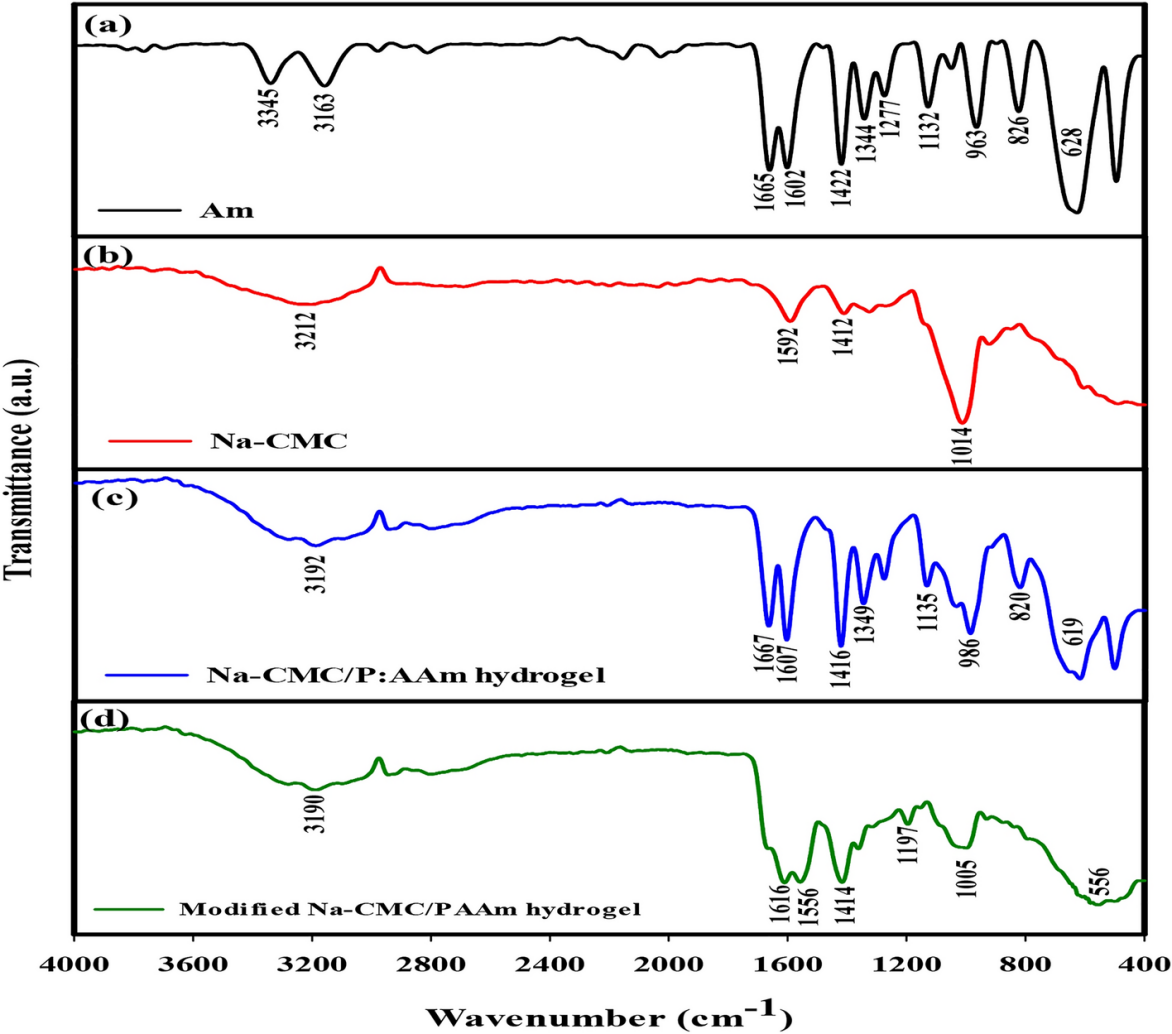
FTIR of Am (**a**), Na-CMC (**b**), Na-CMC/PAAm hydrogel (**c**), and modified Na-CMC/PAAm hydrogel (**d**).

From Fig. 1, the FTIR spectra of the various components and hydrogel products revealed their chemical characteristics and possible interconnectivity. Acrylamide (Am) spectrum (Fig. 1a): The wide hump around 3200 cm^−1^ is due to the N–H stretching of the amide linkage^22^. The bands at 1670 cm^−1^ and 1610 cm^−1^ are related to the C=O and N–H vibrations in Am due to the amide group of the polymer^23^. The split band at 3212 cm^−1^ in Fig. 1b corresponds to the O–H stretching of hydroxyl groups in CMC. The important bands or peaks for this compound are at around 1592 cm^−1^ which are due to carboxylate (–COO–) anion stretching. The peak found at 1412 cm^−1^ was due to the methyl group flexural motion and the ether linkages (C–O–C) in the cellulose backbone were due to 1014 cm^−1^. The general 3000–3500 cm^−1^ peak in Fig. 1c is due to the N–H groups from the polyacrylamide chain present in the hydrogel. The 1667 cm^−1^ and 1607 cm^−1^ bands are attributed to the C=O stretch and N–H bond of the amide groups which Am has added to the hydrogel network^24^. In modified C=O and N–H, they go slightly low to 1616 cm^−1^ and 1556 cm^−1^ respectively in Fig. 1d^25^.

### The swelling percentage and gel fraction of Na-CMC/PAAm hydrogel

**Fig. 2:**
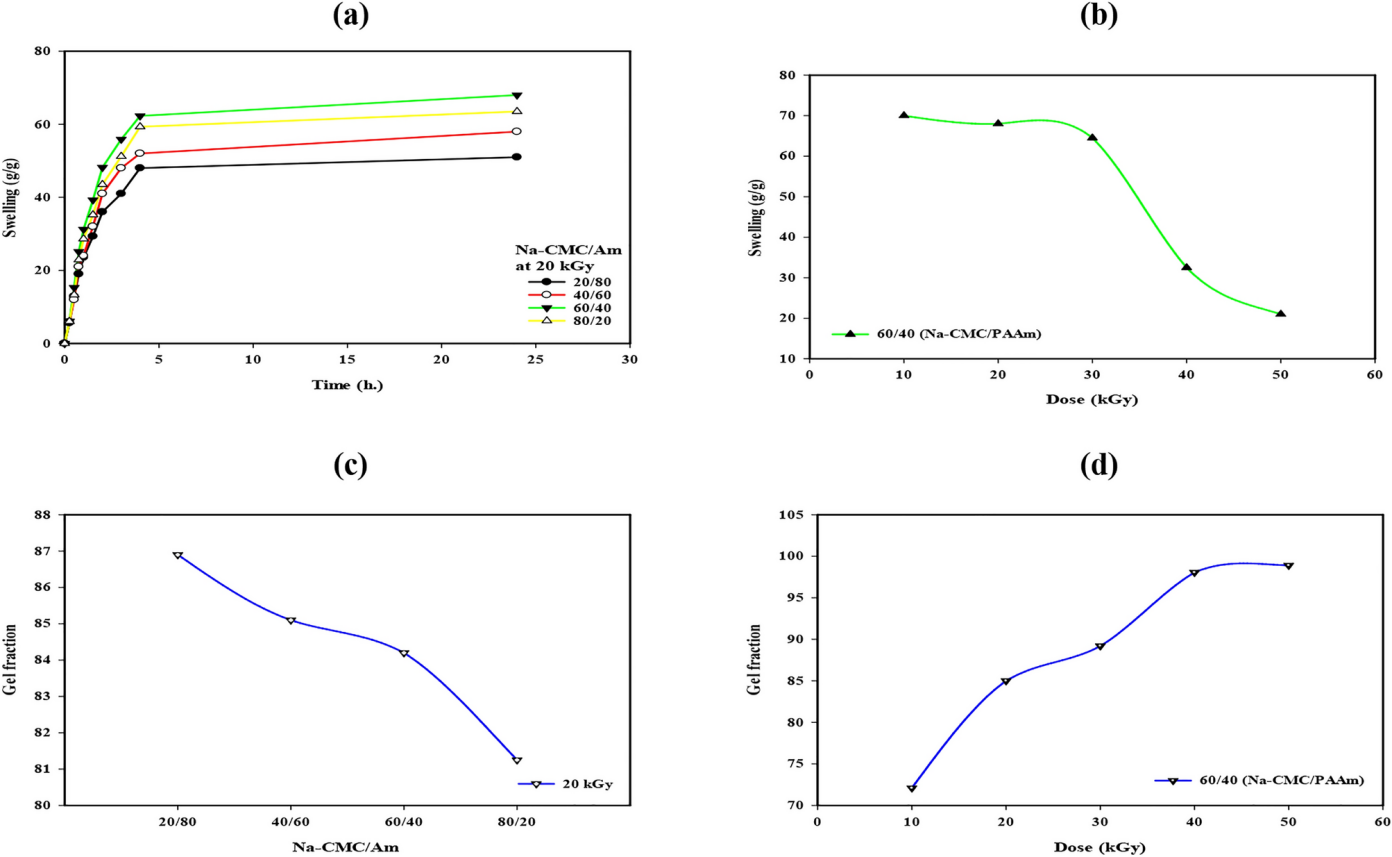
The swelling ratio (g/g) of Na-CMC/PAAm hydrogels at different ratios of Na-CMC/Aam (**a**) and at different radiation doses (**b**), and the gel fraction of Na-CMC/PAAm hydrogels at different ratios (**c**) and at different radiation doses (**d**).

As shown in Fig. 2a and b, the evaluation of swelling ratio in terms of g per g demonstrated that an increase in the amount of acrylic acid incorporated has corresponding swelling rates that reach the maximum value at the 60/40 ratio. Further changes in the acrylamide percentage cause a noticeable reduction in the swelling rates due to changes in crosslinking density and chain conformations within the hydrogel network. More crosslinking density are added to the hydrogel network as the acrylamide concentration increases. The network’s capacity to absorb and hold water is limited by this denser crosslinked structure, which results in less swelling. Even though acrylamide is hydrophilic, the hydrogel’s ability to expand is ultimately constrained by the tighter network creation. This is a rather complex response that highlights the need to align the hydrogel parameters to realize the desired characteristics for the intended application. Particularly, a slight reduction in swelling rates is seen with the use of radiation doses 10–30 kGy which is explainable by slight changes in crosslinking densities and network dynamics. Nevertheless, an increase in the radiation doses to 40 and 50 kGy leads to a considerable decrease in the swelling rates.

In the same context, it was also important to determine the effect of the ratio of the hydrogel constituents Na-CMC/Am together with the radiation doses on the gel fraction rate as described in Fig. 2c and d. There is now a distinguishable trend depicting the relationship between the composition of the hydrogel and the degree of radiation exposure and gel fraction. Interestingly, an increase in acrylamide concentration and radiation dose gives the maximum gel fraction. As the natural polymer content (Na-CMC) increases, the gel fraction decreases primarily due to the reduced crosslink density within the network. Natural polymers, introduce flexible, hydrophilic segments that hinder the efficient formation of crosslinks with synthetic monomers. This disruption limits the extent of network formation and reduces the gel fraction^26^.

### Modification process and water retention of Na-CMC/PAAm hydrogels

**Fig. 3:**
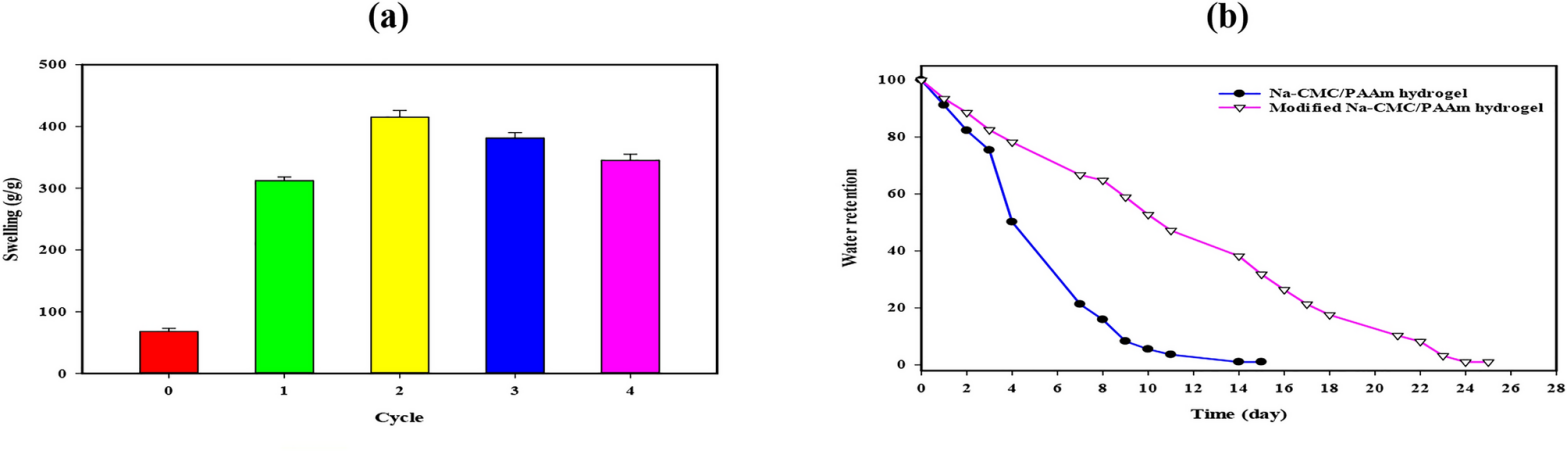
The swelling ratio (g/g) of Na-CMC/PAAm hydrogels at different modification cycles bt NaOH (**a**) and the water retention % at different times of Na-CMC/PAAm and modified Na-CMC/PAAm hydrogels (**b**).

The swelling rate surged to 312 (g/g) during the initial cycle, reaching 415 (g/g) during the second cycle before gradually tapering to 345 (g/g) by the fourth cycle (Fig. 3a). As shown in Fig. 3b the water retention of the prepared hydrogels. The blank hydrogel exhibits a notable loss of 25% of its water content within three days. Also, it demonstrates accelerated water loss, with a staggering 50% reduction in water content occurring approximately five days post-preparation; conversely, the Modified hydrogel presents a stark departure, showcasing prolonged water retention kinetics. A modest 25% water loss is observed after five days, with 50% depletion occurring after ten days and a substantial 75% loss occurring around 16 days. Notably, it takes 23 days for the Modified hydrogel to release its water content fully.

### The SEM analysis of (HEC/PAAm) hydrogel samples

**Fig. 4:**
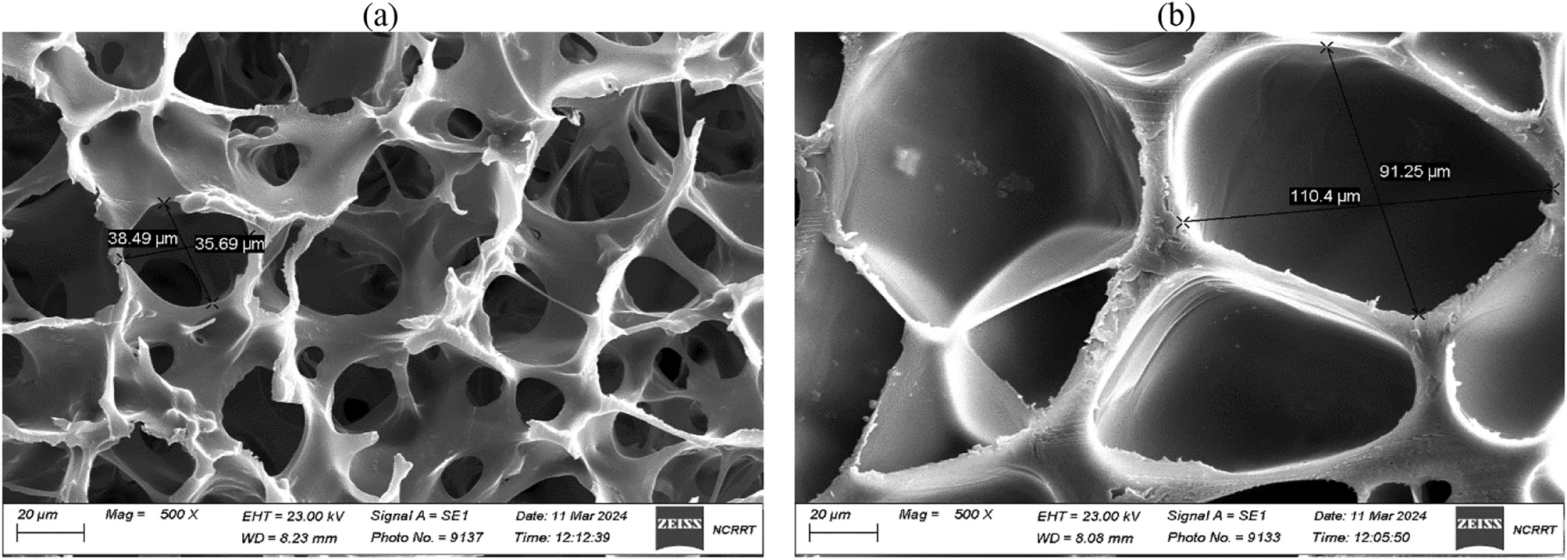
SEM of Na-CMC/PAAm (**a**) and modified (**b**) hydrogels.

The SEM micrographs in Fig. 4 provide valuable insights into the structural transformations induced by the KOH modification process on the Na-CMC/PAAm hydrogels. The unmodified hydrogel (Fig. 4a) exhibits a uniform surface topography characterized by shallow, evenly distributed pores of consistent size and morphology. In contrast, the modified hydrogel (Fig. 4b) displays a strikingly different surface architecture, featuring an array of substantially larger cavities and pores with varying dimensions.

### Influence of varied water stress levels on morphological and yield characteristics of sugar beet using superabsorbent hydrogel (SAH)

**Fig. 5:**
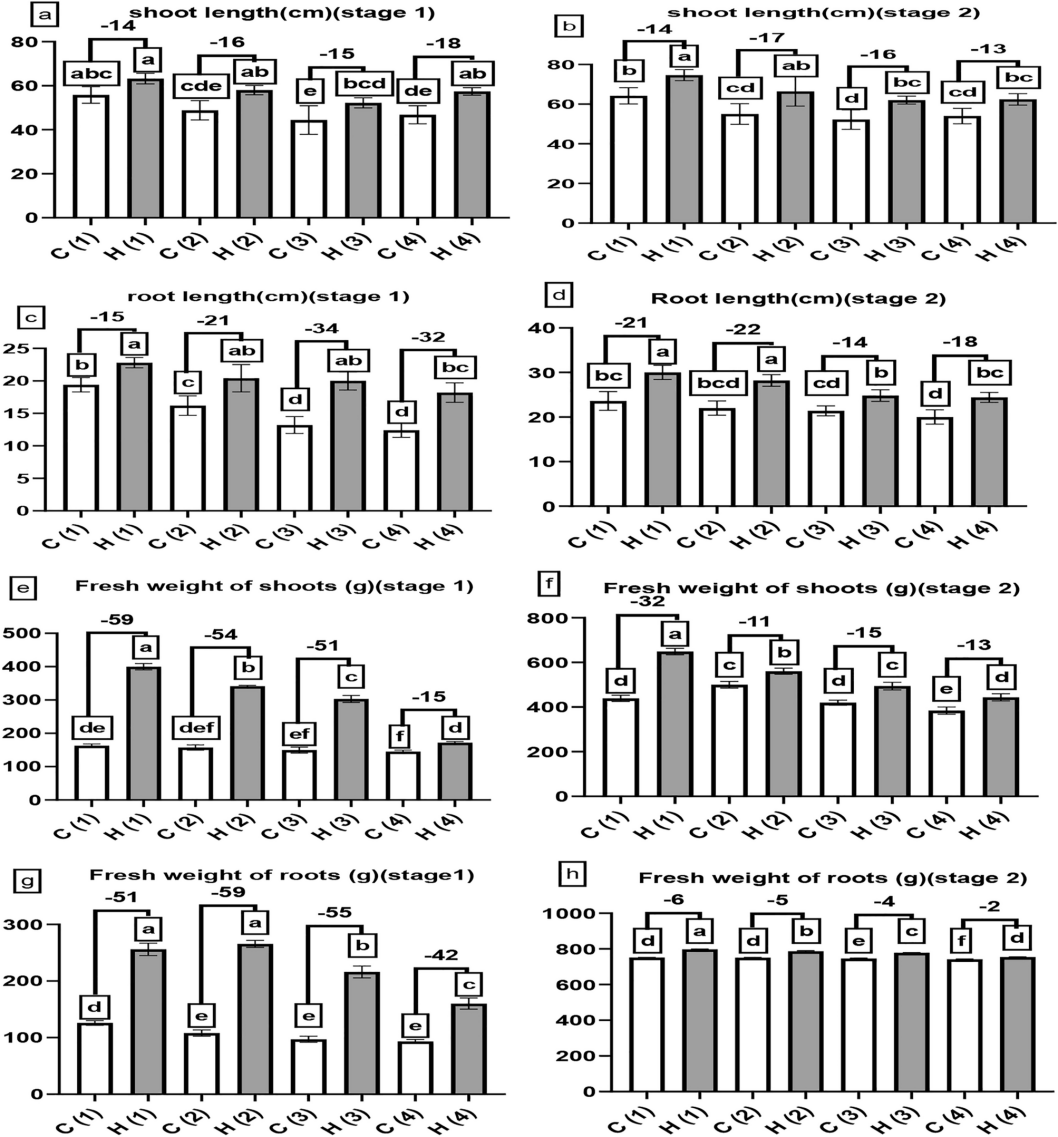
The influence of different levels of water drought stress (0%, 25%, 50%, and 100%) on the morphological characteristics of *Beta vulgaris* at two growth stages: stage 1 (after 60 days of cultivation) and stage 2 (after 90 days of cultivation). The following parameters were evaluated: (**a**) and (**b**) represented the shoot length (in cm), (**c**) and (**d**) denoted the root length (in cm), (**e**) and (**f**) indicated the fresh weight of shoots (in grams) and (**g**) and (**h**) indicated the fresh weight of roots (in grams). In the graphical representation, the control group (without hydrogel) is illustrated by white columns, while the hydrogel-treated group is depicted by gray columns. Statistical analysis (*P* < 0.05) was conducted by ANOVA, and the study involved a sample size of n = 6.

Figure 5 On the data represented in (a) and (b) is quite different on the other hand sugar beet shoot increased length by 14, 16, 15, and 18% at 0, 25, 50, and 100% of drought stress at stage 1 by using SAH in comparison to control treatments, the highest shoot length was obtained in treatment (H1) (63.2 cm) since the lowest one was given in treatment (C3) (44.4 cm), no significant difference found between C1 and H4 at stage 1, moreover, during stage 2 SAH caused a growth in the shoot length by 14, 17, 16, and 13% at 0, 25, 50, and 100% stress respectively, the highest exact reading was (H1) (74.6 cm) while the lowest one is (C3) (52.2 cm), also no significant difference found between C1 and H4 at stage 2, the root lengths observed in Fig. 5c and d that were helped increased by 15, 21, 34, and 32% at 0, 25, 50, and100% stress at stage 1 by using SAH, the longest root length was (H1) (22.8cm) and the shortest being (C4) (12.4 cm), no difference was found between C1 and H4 on root lengths at stage 1, nevertheless, at stage 2 the root length was increased by 21, 22, 14, and 18% at 0, 25, 50, and 100% stress, the highest treatment was (H1) (30cm) and the lowest was (C4) (20cm), also no difference was found between C1 and H4 on root lengths at stage 2.

**Fig. 6:**
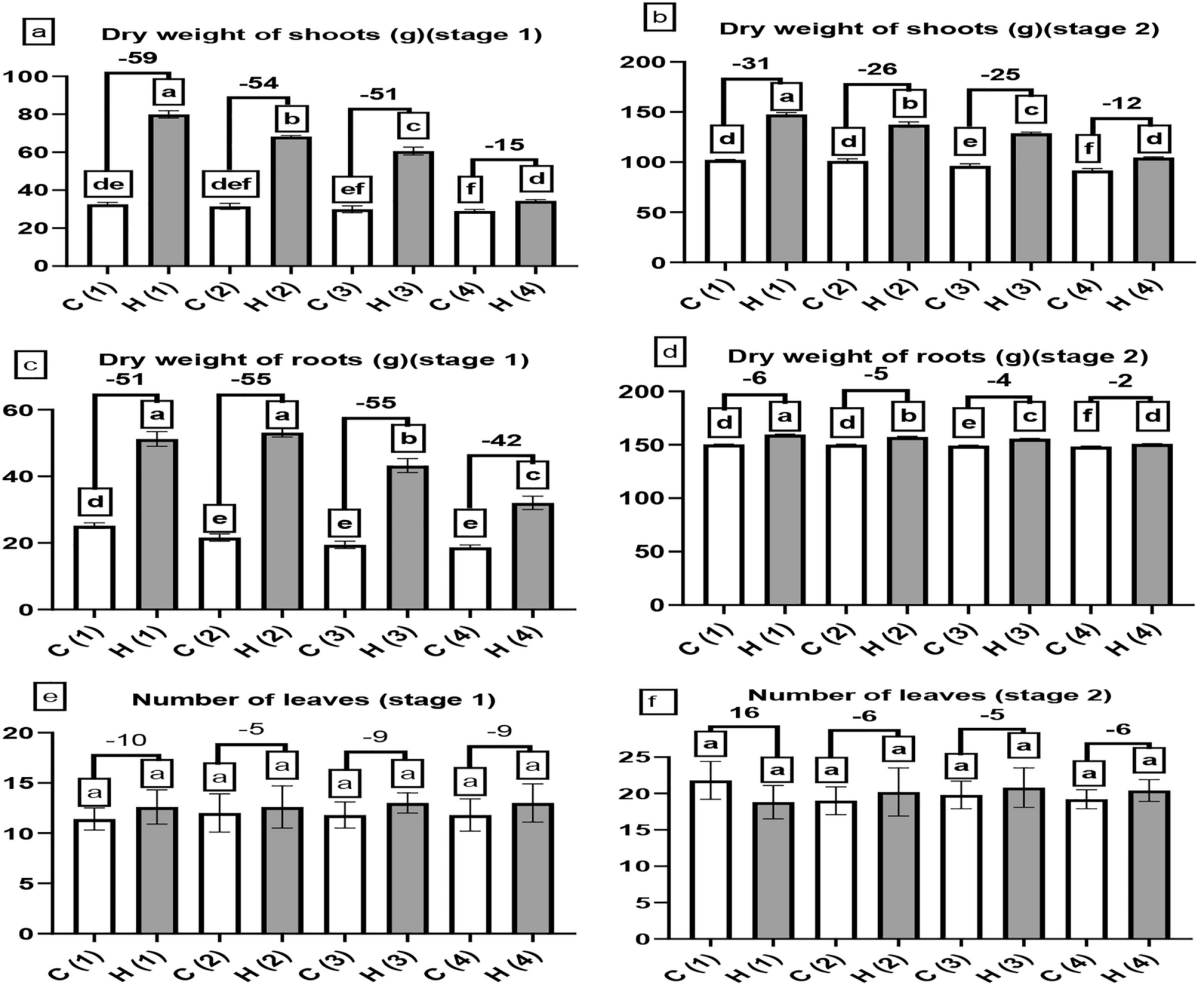
The influence of different levels of water drought stress (0%, 25%, 50%, and 100%) on the morphological characteristics of Beta vulgaris at two growth stages: stage 1 (after 60 days of cultivation) and stage 2 (after 90 days of cultivation). The following parameters were evaluated: (**a**) and (**b**) represented the dry weight of shoots (in grams), (**c**) and (**d**) denoted the dry weight of roots (in grams), and (**e**) and (**f**) indicated the number of leaves. In the graphical representation, the control group (without hydrogel) is illustrated by white columns, while the hydrogel-treated group is depicted by gray columns. Statistical analysis (*P* < 0.05) was conducted by ANOVA, and the study involved a sample size of n = 6.

**Fig. 7:**
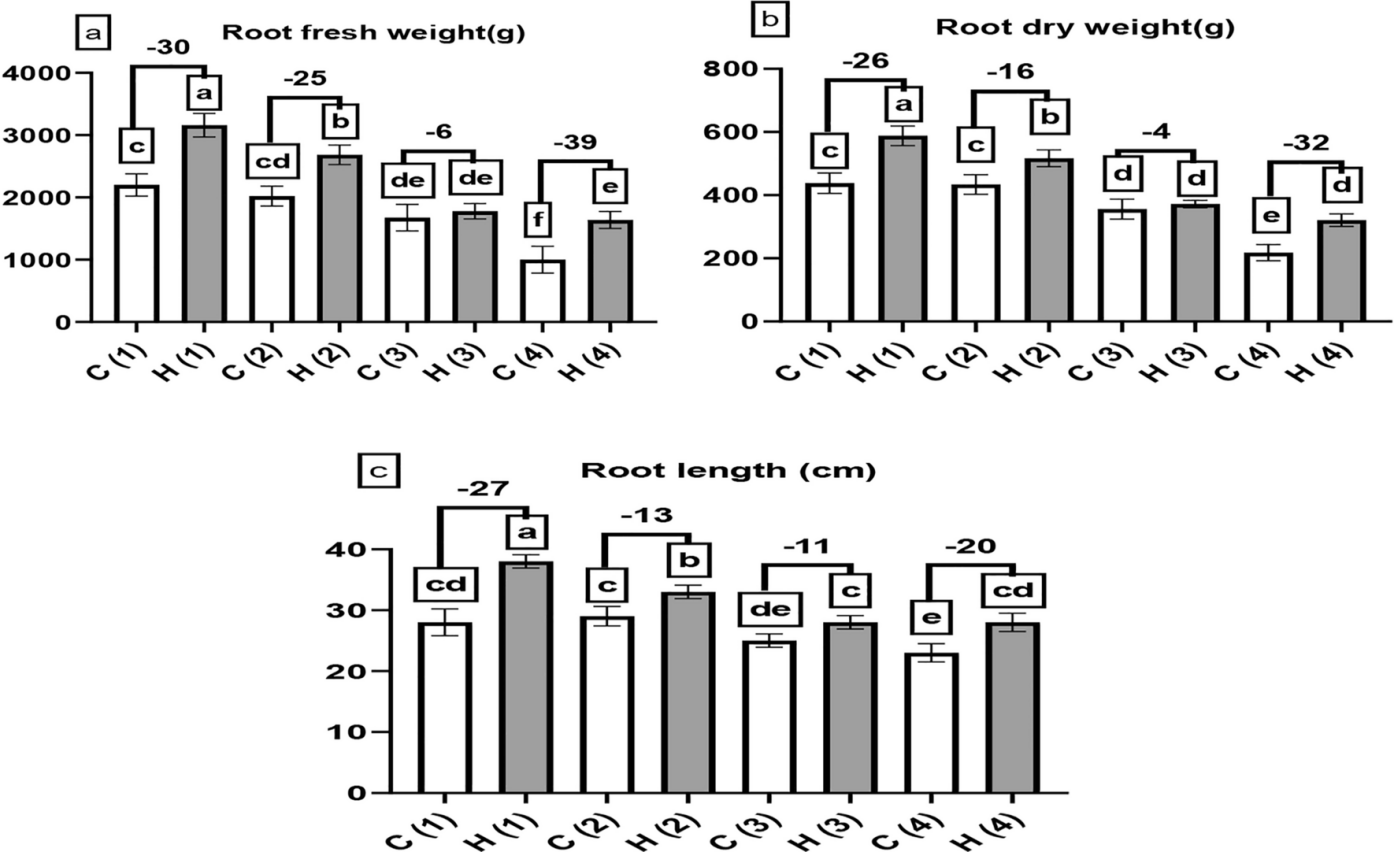
The influence of different levels of water drought stress (0%, 25%, 50%, and 100%) on the yield parameter of *Beta vulgaris*. The following parameters were evaluated: (**a**) represented the root fresh weight (in gram), (**b**) denoted the root dry weight (in grams) and (**c**) denoted the root length (cm). In the graphical representation, the control group (without hydrogel) is illustrated by white columns, while the hydrogel-treated group is depicted by gray columns. Statistical analysis (*P* < 0.05) was conducted by ANOVA, and the study involved a sample size of n = 6.

To start with fresh weight of shoots indicated in Fig. 5e and f SAH being exposed to a drought stress of 59, 54, 51 and 15% respectively at 0, 25, 50, 100% at stage 1 has caused the biggest change, the highest number of reading of shoot fresh weight is with H1 (399.5 g) that obtained the least one with C4 (145.4 g), similarly, also there is a considerable improvement of shoot fresh weight at stage 2 increased by 32, 11, 15 and 13% respectively at 0, 25, 50, 100% at stage 2, the highest number of reading of shoot fresh weight is with H1 (648.8 g) that obtained the least one with C4 (384 g),there are slight increase in shoot fresh weight at H4 treatment when compared to C1 at both stages, In line with the statistical results we have gathered earlier, it appears that SAH symptomatic fresh roots weights got boosted at both stage, there are significant enhancement in root fresh weight at H4 treatment when compared to C1at stage 1 but no difference found at stage 2,

Figure 6a and b, it recorded shoot dry weight progress by SAH indeed Fig. 6a and b, in phase 1 increased by 59, 54, 51 and 15% respectively at 0, 25, 50, 100%,the highest read was tagged to H1(79.96) and the lowest one is tagged to C4 (29.08), in phase 2 SAH delayed the deterioration of un-treated plants compared to those not treated, shoot dry weight increased by 31, 26, 25 and 12% respectively at 0, 25, 50, 100%, the greatest reading value was recorded in H1 (147.56) and the least one was in C4 (91.8), there are slight increase in shoot dry weight at H4 treatment when compared to C1 at both stages, also, the root dry weight is displayed in Fig. 6c and d elevate due to SAH in step 1 by 51, 55,55 and 42% at 0, 25, 50 and 100% stress, the highest read recorded in H2 (53.12) the lowest one in C4(18.68), at stage 2 SAH was significantly better than the controls, there are significant enhancement in root dry weight at H4 treatment when compared to C1at stage 1 but no difference found at stage 2. In Fig. 6e there are slight increase in number of leaves at H4 treatment when compared to C1 at stage 1. Number of leaves is displayed in Fig. 6e elevate due to SAH in stage 1 by 10, 5, 9 and 9% at 0, 25, 50 and 100% stress, the highest value recorded in H1 (10%). During stage 2 in Fig. 6f, the highest value of number of leaves appeared with control group (C1). Due to these improvements in the results, yield had to be better with the treatment of SAH as is shown in Fig. 7a–c, fresh weight rose considerably in the presence of SAH by 30, 25, 6 and 39% at 0, 25, 50 and 100% stress, the major reading recorded in H1 (3.16 kg) while the least in C4 (1 kg).

Due to these improvements in the results, yield had to be better with the treatment of SAH as is shown in Fig. 7a–c, fresh weight rose considerably in the presence of SAH by 30, 25, 6 and 39% at 0, 25, 50 and 100% stress, the major reading recorded in H1 (3.16 kg) while the least in C4 (1 kg).

### Study the influence of (SAH) on the protein, carbohydrates, proline, and phenolic compounds in sugar beet under various stress levels (0%, 25%, 50%, and 100%), comparative to the control treatments

**Fig. 8:**
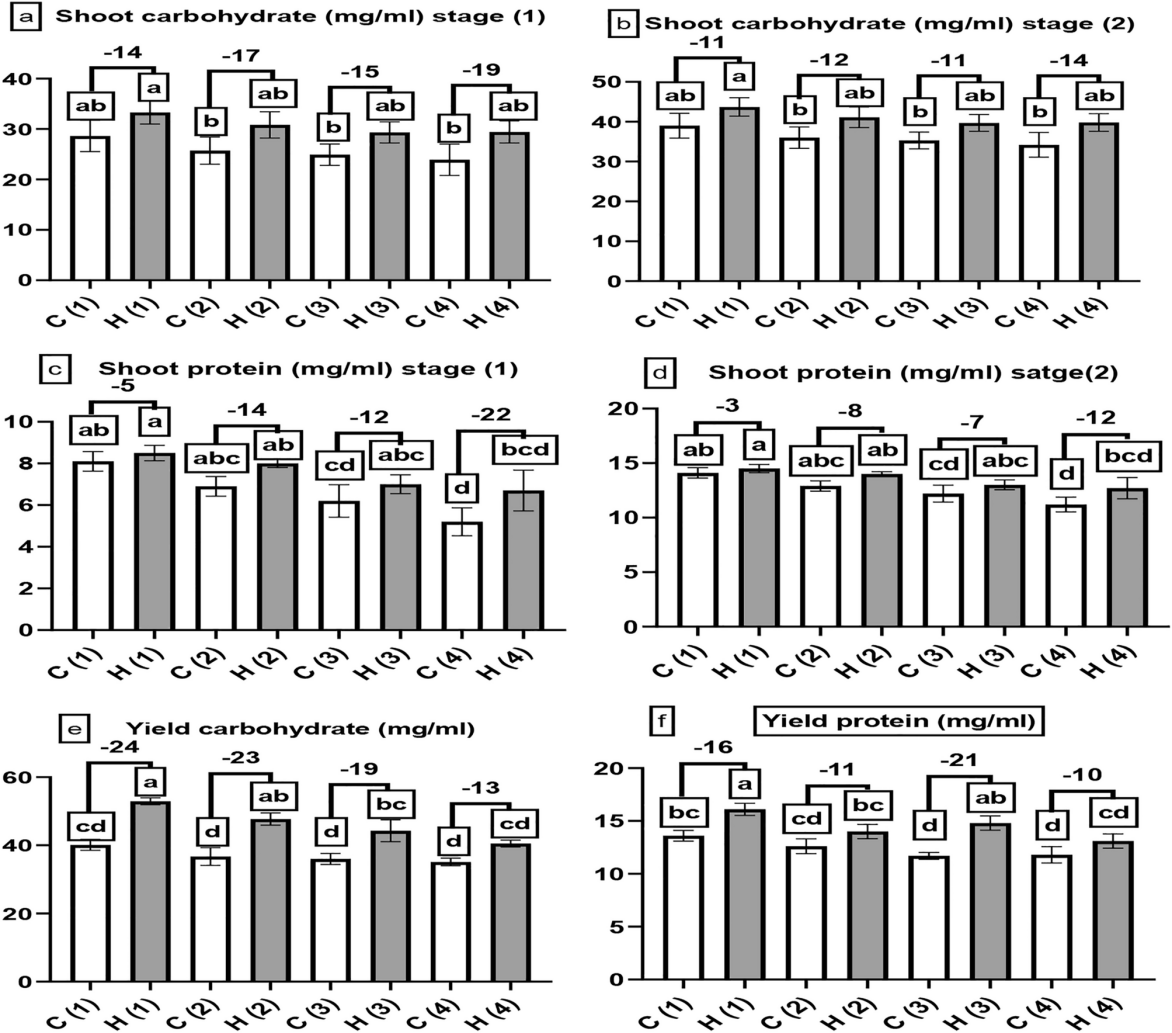
The influence of different levels of water drought stress (0%, 25%, 50%, and 100%) on the shoot and yield analysis of Beta vulgaris at two growth stages: stage 1 (after 60 days of cultivation) and stage 2 (after 90 days of cultivation). The following parameters were evaluated: (**a**) and (**b**) represented the shoot carbohydrate (mg/ml), (**c**) and (**d**) denoted the shoot protein (mg/ml), (**e**) denoted the yield carbohydrate (mg/ml) and (**f**) indicated the yield protein (mg/ml). In the graphical representation, the control group (without hydrogel) is illustrated by white columns, while the hydrogel-treated group is depicted by gray columns. Statistical analysis (*P* < 0.05) was conducted by ANOVA, and the study involved a sample size of n = 6.

**Fig. 9:**
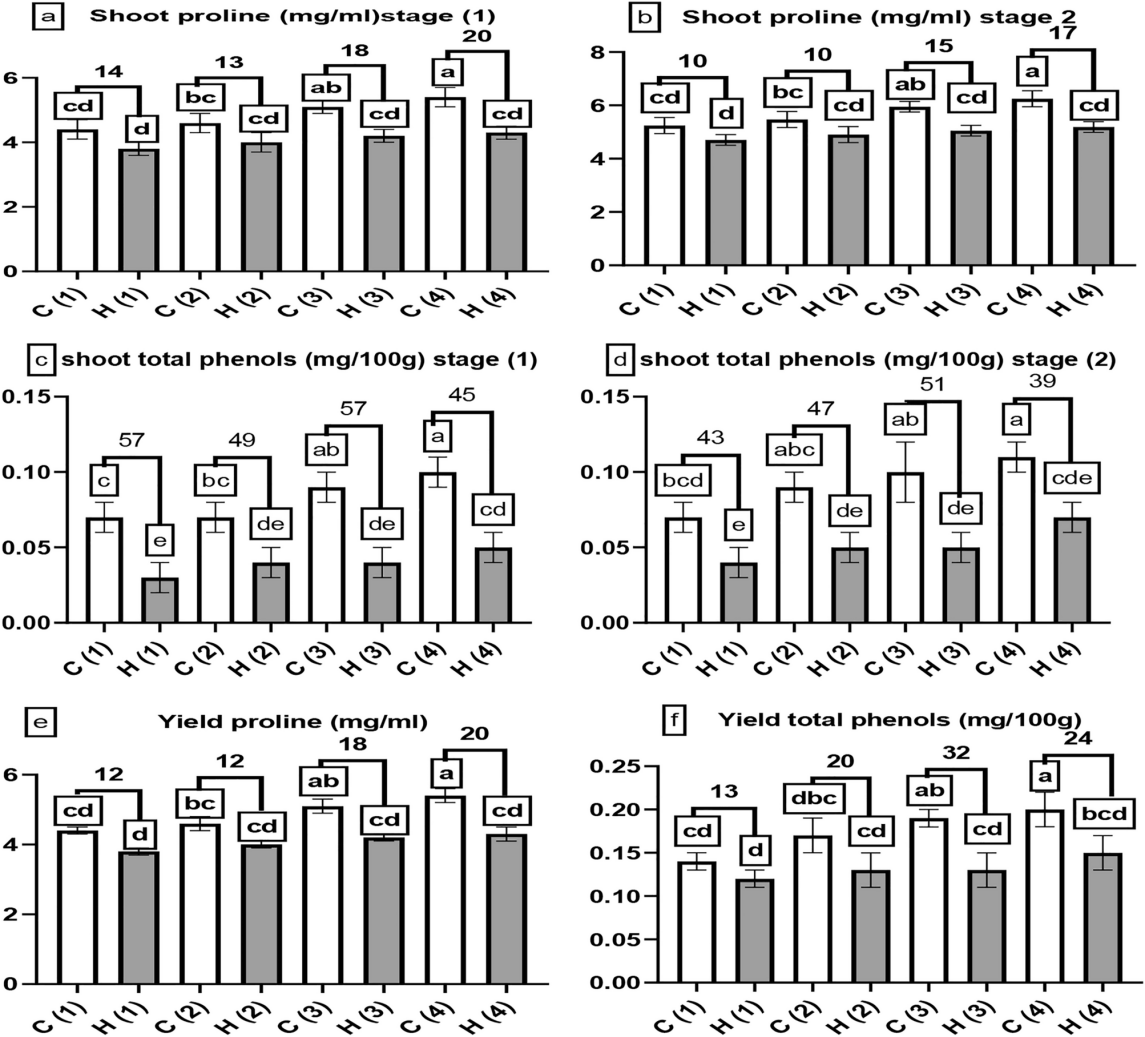
The influence of different levels of water drought stress (0%, 25%, 50%, and 100%) on the shoot and yield analysis of Beta vulgaris at two growth stages: stage 1 (after 60 days of cultivation) and stage 2 (after 90 days of cultivation). The following parameters were evaluated: (**a**) and (**b**) represented the shoot proline (mg/ml), (**c**) and (**d**) denoted the shoot phenol (mg/100 g), (**e**) denoted the yield proline (mg/ml) and (**f**) indicated the yield phenol (mg/100 g). In the graphical representation, the control group (without hydrogel) is illustrated by white columns, while the hydrogel-treated group is depicted by gray columns. Statistical analysis (*P* < 0.05) was conducted by ANOVA, and the study involved a sample size of n = 6.

The data displayed in Fig. 8a, b, and e agreed with the previous concept. They revealed that SAH significantly enhanced carbohydrate content on sugar beet at stage 1 and stage 2 and yield at stage 1; SAH enhanced carbohydrate level by 14, 17, 15 and 19% at 0, 25, 50 and 100% stress when compared to controls, the highest reading was found in H1 (33.3 mg/ml). In comparison, the lowest one was recorded in C4 (23.9 mg/ml), no difference was presented between C1 and H4 treatments at both stages, at stage 2, SAH enhanced carbohydrate level by 11, 12, 11 and 14% at 0, 25, 50 and 100% stress when compared to controls, the highest reading was found in H1 (43.7 mg/ml), while the lowest one recorded in C4 (34.2 mg/ml) also in yield, SAH enhanced carbohydrate level by 24, 23, 19 and 13% at 0, 25, 50 and 100% stress when compared to controls, the highest reading was found in H1 (52.9 mg/ml) while the lowest one recorded in C4 (35.1 mg/ml), no differences noticed between C1 and H4 treatments. Also data presented in Fig. 8c, d and f indicated that SAH have a superiority when compared to controls at stage 1, stage 2 and yield in sugar beet protein content, at stage 1, SAH enhanced protein level by 5, 14, 12 and 22% at 0, 25, 50 and 100% stress when compared to controls, the highest reading recorded at H1 (8.5 mg/ml) while the lowest one recorded at C4 (5.2 mg/ml), no differences noticed between C1 and H4 treatments, at stage 2, SAH enhanced protein level by 3, 8, 7 and 11% at 0, 25, 50 and 100% stress when compared to controls and no differences noticed between C1 and H4 treatments, the highest reading recorded at H1 (14.5 mg/ml) while the lowest one recorded at C4 (11.2 mg/ml) also at yield, SAH enhanced protein level by 16, 11, 21 and 10% at 0, 25, 50 and 100% stress when compared to controls and no differences was found between C1 and H4 treatments, the highest reading recorded at H1 (16.1 mg/ml) while the lowest one recorded at C4 (11.8 mg/ml),

Figure 9a, b and e displayed that SAH decreased proline content when compared to controls, at stage 1 proline content decreased by 14, 13, 18 and 20% at 0, 25, 50 and 100% stress, at stage 2 proline content decreased by 10, 10, 15 and 17% at 0, 25, 50 and 100% stress also yield proline content was decreased by 12, 12, 18 and 20% at 0, 25, 50 and 100% stress, no differences was found between C1 and H4 treatments, phenol content decreased by 57, 49, 57 and 45% at 0, 25, 50 and 100% stress at stage 1 and decreased by 43, 47, 51 and 39% at 0, 25, 50 and 100% stress at stage 2 also yield phenol decreased by 13, 20, 32 and 24% at 0, 25, 50 and 100% stress, no differences was found between C1 and H4 treatments.

### Study the influence of (SAH) on chlorophyll and carotenoid content in sugar beet under various stress levels (0%, 25%, 50%, and 100%) compared to the control treatments

Agreeing with the previous data our results presented in Table 3 showed that SAH improved chlorophyll a by 16 and 13 at 25 and 50% stress but no difference was found at 0 and 100% stress, the highest recorded in H1 and C1 (12.8) and the lowest one recorded in C4 (8.2) at stage 1, while at stage 2, SAH enhanced chlorophyll a level by 9, 15, 12 and 5% at 0, 25, 50 and 100% stress when compared to controls, H1 recorded (14.7) and C4 recorded (9.7), in continuation to our data chlorophyll b increased significantly by using SAH at 0 and 100% stress by 20 and 26%, H1 is the highest record (5.9) and C4 is the lowest (2.9) at stage 1, at stage 2, chlorophyll b increased significantly by using SAH at 0 and 100% stress by 17 and 11%, the highest reading recorded in H1 (8.8) and the lowest in C4 (6.0),no difference was noticed between C1 and H4, also total chlorophyll enhanced by using SAH as compared to controls by 6, 6, 3 and 9% at 0, 25, 50 and 100% stress at stage 1 and 12, 3, 3 and 7% at 0, 25, 50 and 100% stress at stage 2, also carotenoid content increased by using SAH, by 21, 30, 23 and 13% at 0, 25, 50 and 100% stress at stage 1 and 23, 34, 29 and 19% at 0, 25, 50 and 100% stress at stage 2, the highest record in H1 (5.2) and the lowest one was recorded in C4 (2.3).

**Table 3:** The influence of (SAH) on chlorophyll and carotenoid content in sugar beet under different levels of water stress.

Treatments	Chlorophyll a (mg/g F. wt.)		Chlorophyll b (mg/g F. wt.)		Total chlorophyll (mg/g F. wt.)		Carotenoids (mg/g F. wt.)	
	Stage (1)	Stage (2)	Stage (1)	Stage (2)	Stage (1)	Stage (2)	Stage (1)	Stage (2)
Control (1)	12.8 ± 0.9a	13.5 ± 0.6bc	4.7 ± 0.2bcd	7.3 ± 0.3cd	17.5 ± 0.69ab	20.7 ± 0.3b	4.1 ± 0.2bc	4.0 ± 1.0bc
Hydrogel (1)	12.8 ± 0.2a	14.7 ± 0.3a	5.9 ± 0.3a	8.8 ± 0.3a	18.7 ± 0.49a	23.5 ± 1.7a	5.2 ± 0.5a	5.2 ± 0.2a
Control (2)	10.2 ± 0.7b	12.0 ± 0.3de	5.4 ± 0.5ab	8.4 ± 0.1ab	15.6 ± 0.24cd	20.4 ± 0.3bc	3.2 ± 0.2de	2.9 ± 0.2de
Hydrogel (2)	12.1 ± 0.6a	14.0 ± 0.6ab	4.5 ± 0.6bcd	7.1 ± 0.6cd	16.6 ± 0.03bc	21.1 ± 1.8b	4.5 ± 0.4ab	4.4 ± 0.3b
Control (3)	9.1 ± 0.4bc	10.8 ± 0.8ef	4.9 ± 0.1bc	7.8 ± 0.4bc	14.0 ± 0.48e	18.6 ± 1.1d	2.6 ± 0.1de	2.5 ± 0.2e
Hydrogel (3)	10.4 ± 0.1b	12.3 ± 0.1cd	4.0 ± 0.2cd	6.8 ± 0.2de	14.4 ± 0.04de	19.1 ± 0.04cd	3.4 ± 0.3cd	3.5 ± 0.1cd
Control (4)	8.2 ± 0.7c	9.7 ± 0.1f.	2.9 ± 0.3e	6.0 ± 0.4e	11.1 ± 0.65f.	15.7 ± 0.4e	2.5 ± 0.1e	2.3 ± 0.2e
Hydrogel (4)	8.3 ± 0.1c	10.2 ± 0.2f.	3.9 ± 0.1d	6.8 ± 0.3de	12.2 ± 0.07f.	17.0 ± 0.2e	2.9 ± 0.2de	2.9 ± 0.1de
HSD at 0.05	0.47	0.38	0.27	0.29	0.37	0.46	0.24	0.18

The mean of six replicates ± standard error of means is represented by each value. Significant differences can be seen between different lowercase letters in the same column.

Values of the same column with the same letter are not substantially different by post hoc-Tukey’s Honestly Significant Difference test (HSD) at *P* ≤ 0.05.

### Study the influence of (SAH) on stress markers (antioxidant enzymes, H_2_O_2_ and MDA) in sugar beet under various stress levels (0%, 25%, 50%, and 100%) compared to the control treatments

The positive effect of SAH, as displayed in the previous results, decreased drought stress of sugar beet; these results agreed with data presented in Table 4, which illustrated that SAH significantly decreased catalase enzyme by 22, 9, 4 and 13% at 0, 25, 50 and 100% stress at stage 1 and 7, 3, 4 and 9% at 0, 25, 50 and 100% stress at stage 2 when compared to control, this may be due to decreasing drought stress and improving overall health. Also, our results showed that SAH decreased peroxidase activity by 10, 11, 15 and 29% at 0, 25, 50 and 100% stress at stage 1and 13, 18, 11 and 28% at 0, 25, 50 and 100% stress at stage 2, no difference was found between C1 and H4; as displayed in the table, the minimum reading was found in H1 at both stages, while C4 gave the highest reading. Also, the results are in our hands, as displayed in Table 4, showed a significant decrease in polyphenol oxidase in SAH treatments compared to controls by 45, 36, 27 and 19% at 0, 25, 50 and 100% stress at stage 1and 21, 16, 9 and 15% at 0, 25, 50 and 100% stress at stage 2, no difference was found between C1 and H4;; the minimum reading was found in H1 treatment at both stages, while the highest was recorded in C4. Data presented in Table 5 revealed that SAH decreased H_2_O_2_ content in plants compared with control treatments by 34, 21, 30 and 30% at 0, 25, 50 and 100% stress at stage 1and 36, 19, 34 and 29% at 0, 25, 50 and 100% stress at stage 2, no difference was found between C1 and H4; the C4 treatment appeared to be the highest reading recorded as exposure to severe drought, while the H1 treatment was the lowest.

**Table 4:** The influence of (SAH) on antioxidant enzymes in Sugar beet under different levels of water stress.

Treatments	Catalase (unit/g. f. wt.)		Peroxidase (unit/g. f. wt.)		Polyphenol oxidase (unit/g. f. wt.)	
	Stage (1)	Stage (2)	Stage (1)	Stage (2)	Stage (1)	Stage (2)
Control (1)	1400 ± 45.8de	1510 ± 17.3cd	186 ± 5.2de	201 ± 10.8cde	43.6 ± 8.2bc	54.4 ± 7.3abc
Hydrogel (1)	1090 ± 62.4f	1400 ± 45.8d	168 ± 7.9e	174 ± 7.9e	24.0 ± 2.4d	43.2 ± 5.2c
Control (2)	1480 ± 34.6cd	1560 ± 30.0bc	219 ± 10.8bc	228 ± 15.9bc	51.6 ± 4.8ab	58.8 ± 7.2ab
Hydrogel (2)	1340 ± 45.8e	1520 ± 17.3c	194 ± 4.6cde	187 ± 7.5de	33.2 ± 8.2cd	49.6 ± 5.7bc
Control (3)	1590 ± 52.0bc	1650 ± 30.0b	243 ± 12.0b	240 ± 19.7b	56.8 ± 6.0ab	58.4 ± 5.0abc
Hydrogel (3)	1520 ± 45.8bcd	1590 ± 30.0bc	207 ± 5.2cd	213 ± 5.2bcd	41.2 ± 4.8bc	53.2 ± 5.0abc
Control (4)	1860 ± 30.0a	1830 ± 79.4a	306 ± 12.0a	312 ± 12.0a	65.2 ± 3.7a	68.4 ± 5.2a
Hydrogel (4)	1620 ± 52.0b	1660 ± 45.8b	216 ± 12.0c	226 ± 6.9bc	52.8 ± 4.8ab	58.0 ± 0.7abc
HSD at 0.05	40.7	35.0	8.01	10.13	4.92	4.76

The mean of six replicates ± standard error of means is represented by each value. Significant differences can be seen between different lowercase letters in the same column.

Values of the same column with the same letter are not substantially different by post hoc-Tukey’s Honestly Significant Difference test (HSD) at *P* ≤ 0.05.

**Table 5:** The influence of (SAH) on H_2_O_2_ and MDA in Sugar beet under different levels of water stress.

Treatments	MDA (mm/g. f. wt.)		H_2_O_2_ (mg/100 ml)	
	Stage (1)	Stage (2)	Stage (1)	Stage (2)
Control (1)	0.3 ± 0.02d	0.5 ± 0.04cd	1.6 ± 0.1bc	3.0 ± 0.1de
Hydrogel (1)	0.3 ± 0.03d	0.4 ± 0.03e	1.1 ± 0.3d	1.9 ± 0.3f
Control (2)	0.5 ± 0.02b	0.5 ± 0.02bcd	1.7 ± 0.1abc	2.9 ± 0.3de
Hydrogel (2)	0.4 ± 0.02c	0.5 ± 0.01de	1.3 ± 0.3cd	2.3 ± 0.4ef
Control (3)	0.6 ± 0.01a	0.6 ± 0.02b	1.9 ± 0.05ab	4.8 ± 0.1b
Hydrogel (3)	0.4 ± 0.03bc	0.5 ± 0.01bcd	1.4 ± 0.1cd	3.1 ± 0.1d
Control (4)	0.6 ± 0.01a	0.7 ± 0.02a	2.1 ± 0.1a	5.5 ± 0.2a
Hydrogel (4)	0.5 ± 0.01b	0.6 ± 0.04bc	1.5 ± 0.1cd	3.9 ± 0.2c
HSD at 0.05	0.02	0.02	0.14	0.19

The mean of six replicates ± standard error of means is represented by each value. Significant differences can be seen between different lowercase letters in the same column.

Values of the same column with the same letter are not substantially different by post hoc-Tukey’s Honestly Significant Difference test (HSD) at *P* ≤ 0.05.

Also, the results presented in Table 5 showed that SAH decreased MDA content in plants under drought stress treatments by 2, 14, 26 and 26% at 0, 25, 50 and 100% stress at stage 1and 15, 8, 11 and 18% at 0, 25, 50 and 100% stress at stage 2, no difference was presented between C1 and H4. Also, the C4 treatment gives the highest recorded number in response to severe drought stress, while the H1 treatment has the lowest reading.

## Discussion

FTIR spectra of the various components and hydrogel products, regarding the structures and interactions as shown in Fig. 1, revealed part of the amide groups were hydrolyzed by KOH, introducing negative charges in the form of carboxylate anions to the hydrogel. The FTIR data attests to the synthesis of the Na-CMC/PAAm hydrogel with the help of gamma radiation to induce CMC & Am monomers’ copolymerization. From the spectra of the resulting modified hydrogel, we can conclude that ionic groups (carboxylates) were grafted into the hydrogel structure after the KOH treatment. The more ionizable –COO– and –NH2 groups are present in the modified hydrogels than in the unmodified hydrogels hence, they are more hydrophilic and capable of absorbing water as obtained from the swelling measurements. This ionic modification as well as the microporosity of the adsorbent enhances the water uptake and its valuable retainment for farms.

Based on present knowledge, the swelling ratio is a key factor used in the evaluation and assessment of the performance of Na-CMC/PAAm hydrogel, a core area of study. The influences of various factors that control the hydrogel swelling behaviour can be described by carrying out precise experiments and applying analytical skills to reveal the fundamental details about the hydrogel you are developing. From the observation of the swelling ratio as depicted in the Fig. 2, the swelling rate increases gradually with time before reaching its maximum swelling at around 24 h of immersion in water. This temporal profile is proof of how the structure of the hydrogel as well as the structure and composition of the surrounding environment affect the overall interaction of the hydrogel. However, the effect of the Na-CMC to acrylamide mass ratio on the swelling characteristics is the most intriguing one. Interesting trends are discovered by methodically changing this ratio, explaining the complex dependency of hydrogel composition on swelling ability. Thus, studying the effect of radiation dosage on swelling behaviour provides useful information concerning the hydrogel’s sensitivity to stimuli. Adjusting the doses of radiation systematically allows for a clear determination of the swelling rates for every dose change, and the variation in hydrogel behaviour with the increase in a radiation dose.

A dramatically lower value of the swelling rates demonstrates a high sensitivity of hydrogel structure and its integrity change. These conclusions show the complex relationship of the external factors, the composition of the hydrogel and its swelling characteristics, and provide a useful guide for the development and optimisation of Na-CMC/PAAm hydrogel for applications in agriculture. The combination of an increase in both acrylamide concentration and radiation dose gives the highest gel fraction by a positive interaction between the two parameters of gel formation. This observation underlines polymers’ propensity to form hydrogels, where changes in the makeup of monomers and the irradiation dose delicately control crosslink formation.

Another modification process influenced the swelling rates of hydrogel formulations that used potassium hydroxide; this process was in harmony with the earlier studies. The first improvement also demonstrated a 75% reduction in blank hydrogel water content within 3 days presented in Fig. 3b which indicated that the water release rate was faster, it was reduced to 50% of its initial value in approximately 5 days which described poor water absorption, The fourth modification was repeated several time and resulted in interesting changes in the trends of swelling motion observed in each successive cycle, This cycle changes emphasize Following the evaluation of the replenishment of water, the various types of hydrogels exhibit separate performance profiles. Thus by taking twice the time taken by the normal hydrogel of 23 days, the Modified hydrogel can release its water content making it relevant to regions that experience dry weather and complications of drought. Therefore, based on the results of this research, it vividly points to the importance of hydrogel modification on the water retention capability, with the Modified hydrogel yielding a good potential in addressing water-scarce situations in drought. After several cycles, the hydrogel retained its basic integrity and functional performance, despite small structural changes caused by the alkaline environment. In particular, no significant degradation that would compromise the structural integrity or effectiveness of the gel was found during the test cycles. The hydrogel was re-allowed to absorb and lose water several times and no significant change was observed.

About the structure in Fig. 4, it can also be deduced that the KOH treatment is responsible for the complete change in pore size and distribution because it promotes the gradual dissolution and growth of the pores on the hydrogel scaffold. These enlarged cavities are formed in hydrogels, an aspect important because it increases the amount of water that is taken by the modified hydrogels. Through the larger pore volume gradient, the hydrogel acquires a higher capacity to incorporate increased volumes of water molecules within the hydrogel structure and thus enhance the extent of imbibition and retention. This structural reconfiguration following KOH modification works hand in hand with the increase in both hydrophilicity and the ionic attraction after the ionization process which has all expounded the impressive water absorption characteristics depicted in the modified hydrogels.

A hydrogel is a material that has the incredible ability to absorb and hold water or other liquids in large quantities^27^; the use of SAH can help sugar beet plants improve drought tolerance^28^; this will lead to the improvement of root sprouting, root growth, and overall growth, in harmony to our results presented in Fig. 5a and b^29^ revealed that SAH highly increased leaf area index of sugar beet, also^29^ proved the positive effect of SAH on root length of sugar beet when compared to non-treated plant agreed with our results presented in Fig. 5c and d, researches recorded in context agreed with our results presented in Fig. 5e and f as^30^ who illustrated the positive effect of super absorbent polymer on *Ficus Benjamina* L. shoot fresh weight in the greenhouse. Consistent with our results presented in Fig. 5g and h^29^ discuss the positive effect of SAH on root fresh weight of sugar beet, this superiority of shoot dry weight by using SAH presented in Fig. 6a and b verified by researches, one of them^29^ who prove the positive effect of SAH on shoot dry weight of sugar beet, these results presented in Fig. 6c and d are consistent with^31^ who revealed that SAH has the appositive effect of sugar beet under drought condition.

The improvement of SAH on sugar beet overall growth led to increased yield production, in harmony with results presented in Fig. 7a–c^32^ displayed the positive effect of SAH on sunflower in the arid and semi-arid region under drought stress, The possible explanation for improvement of morphological and yield parameter by using SAH may by improving water retention in the soil, improving root development, enhancing leaf growth, better nutrient uptake and reducing stress symptoms, which can be beneficial in arid or drought-prone environments.

During periods of drought, crop photosynthesis rates decline due to the simultaneous occurrence of stomatal and non-stomatal limitations; under severe stress, non-stomatal factors play a more significant role in reducing photosynthesis, whereas in mild drought conditions, stomatal limitations predominantly affect net photosynthesis^33,34^. It is possible to increase carbohydrate levels in plants by reducing drought stress and maintaining soil moisture which enhances photosynthesis and generates more carbohydrates; such as our data presented in Fig. 8a, b^35^ which showed that, SAH can enhance carbohydrate content in plants by reducing drought stress and maintaining soil moisture, leading to enhanced photosynthesis and carbohydrates; the same approach of our data presented in Fig. 8a, b^35^ illustrated the positive effect of SAH on soybean grains soluble carbohydrates content under drought conditions in Brazilian Cerrado. SAH may contribute to more efficient water and nutrient uptake by preventing water and nutrient leaching^36^; this can lead to an increased supply of nitrogen (an essential component for protein production in plants); the results in Fig. 8c, d are consistent with^37^ who showed the positive effect of SAH on protein content of tolerant tree species under drought condition in semi-arid region. The ability of the SAH to hold water and slowly release it into the plant roots can help sugar beet plants maintain adequate water levels even in dry weather^28^.

**Fig. 10:**
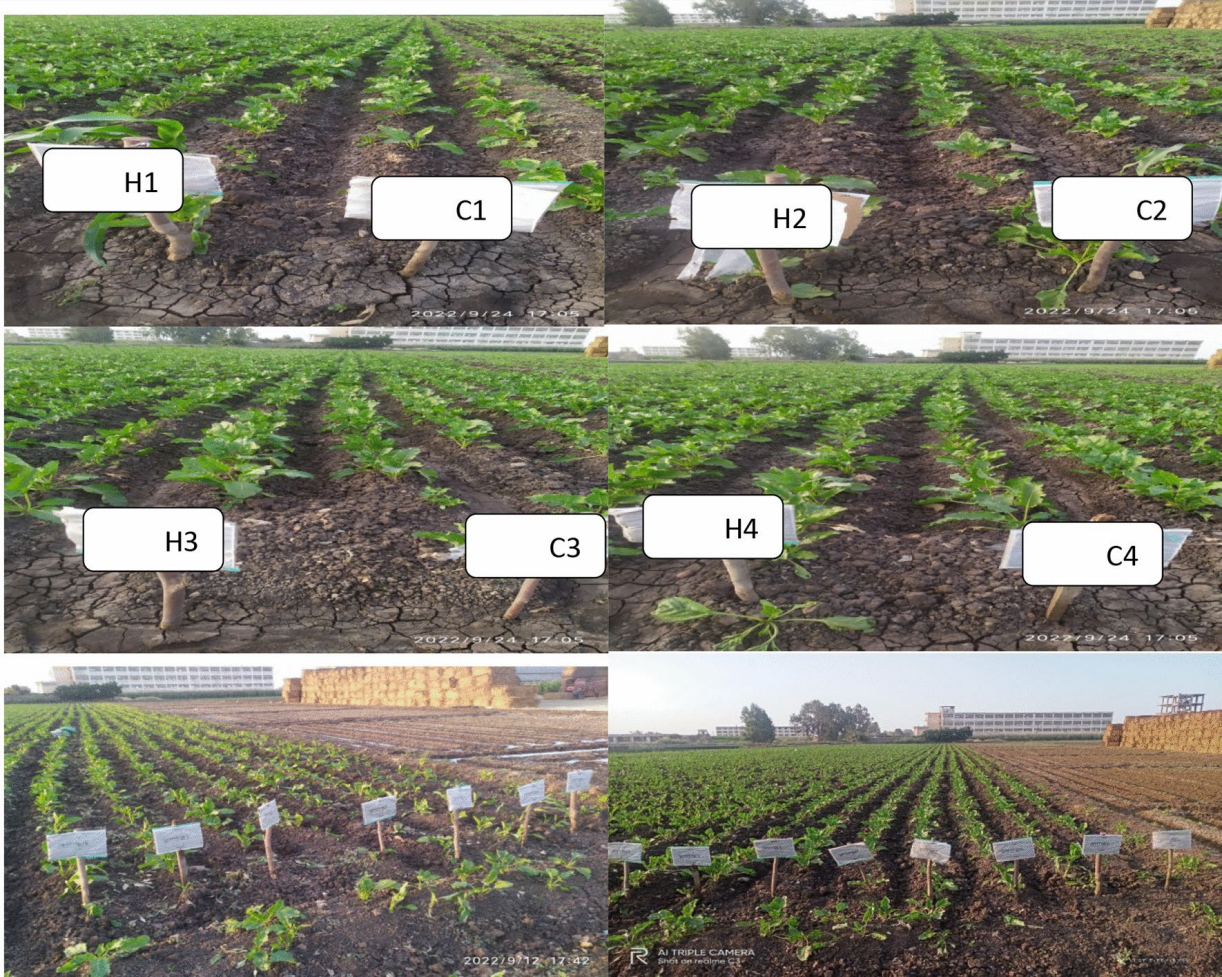
Utilization of UAH on *Beta vulgaris* (sugar beet) at Various Stress Levels (0% (**1**), 25% (**2**), 50% (**3**), and 100% (**4**)). The control samples (without hydrogel) are denoted by the letter (C), while the hydrogel-enhanced samples are represented by the letter (H).

Proline accumulation may decrease due to decreased drought stress because proline is usually synthesized in response to water stress^38^, in line with our results presented in Fig. 9a, b and e^39^ showed that SAH gives a significant reduction of proline content in maize plant. When plants experience drought stress, they may produce phenolic compounds to protect themselves from different environmental stressors^40^; SAH has shown promising results in reducing the impact of drought stress on sugar beet plants. A noticeable decline in phenolic compounds, which are harmful to plants, is a result of this. The breakthrough may have the potential to improve significantly resilience in sugar beet crops and assist farmers in fighting drought effects^41^. This concept agreed with our results presented in Fig. 9c, d and f which displayed that SAH gives a significant reduction in phenol content when compared to control treatments, The results in Fig. 9c, d and f are consistent with the findings of^42^ that cellulose-based hydrogel could reduce drought stress on sunflower plant which is harmful and decrease phenol content (Fig. 10).

Having enough water is essential for plants to carry out photosynthesis, a process that allows them to produce chlorophyll and carotenoids^43^, SAH, by ensuring consistent water availability, may mitigate the negative impact of drought on chlorophyll levels, helping to maintain photosynthetic activity^44^, the noticeable improvement in chlorophyll and carotenoid content may be due to the following:, SAH can help retain soil moisture, reducing the severity of drought stress, Adequate water availability supports photosynthesis, help maintain a more favorable environment for plant metabolism, minimizing oxidative damage and preserving the pigments involved in photosynthesis and SAH can enhance nutrient retention in the soil, ensuring that essential nutrients for chlorophyll and carotenoid synthesis are available to the plants, our results presented in Table 3 support our previous research on Poly(Starch/Acrylic Acid) Superabsorbent Hydrogel on sunflower plant under drought condition that ensured the positive effect of hydrogel on chlorophyll and carotenoid content^45^.

Plants require sufficient water to carry out photosynthesis; it is also this process that results in the production of chlorophyll and carotenoids by constantly supplying water. Thus, SAH might be helping in mitigating the adverse effect of drought on the pigment content at a more considerable level. Moreover, the significant increase in the chlorophyll and carotenoid contents can be attributed to the following reasons; SAH withholds soil moisture as such minimizes the adverse effects of drought stress, in addition, sufficient water is needed to keep the process of photosynthesis moving. It also buffers the plant in a friendlier manner for its metabolism, hence minimizing the oxidative damage and therefore protecting the pigments involved in photosynthesis. SAH is used to improve nutrient holding power in the soil always assuring that micro and macro elements required for chlorophyll and carotenoid synthesis will be made available to the plant. Our results reported in Table 4 support our previously reported results for Poly(Starch/Acrylic Acid) Superabsorbent Hydrogel on sunflower plants under drought conditions that ensured the positive effect of hydrogel on chlorophyll and carotenoid content of the plant^45^. For example, drought stress leads to the overproduction of reactive oxygen species (ROS) in plants. These can further affect cells through oxidative damage.

On the other hand, the antioxidant enzymes come into action by scavenging these dangerous molecules and helping the plant in dealing with the stress. According to research by Sachdev et al.^46^ the requisite of the major antioxidant enzyme in serviceable hydrogen peroxide scavenging in plants is catalase. In contrast to this, we found in our results, as shown in Table 4, in conformity with the results of Ahmed et al.^47^ who used natural polymer hydrogel to lessen the drought stress impact of tomato, the part played in hydrogen peroxide application by peroxidase, which is an enzyme catalase, that catalyzes oxidations of various substrates, while, on the other hand, it has another similar function that has an important role in many physiological processes. Oxidation of peroxidase functions is among the most important kinds of defence processes in the event of stresses on plants and fights against pathogens. On the other hand, being a defence process by nature, it is mostly slow to respond to agents of stress. Mechanisms of actions are large because of the increase in the water retention in the soil, increasing the nutrient availability in the root zone, the availability of water with the node of the time and the node of the amount of it, so usual healthy physiological processes can be developed upon the same approach used by Gomaa and Aldaby^48^ who used hydrogel to lessen catalase and peroxidase of sunflower under drought stress. PPO is the peroxidase–another plant enzyme that uses an intermediate substrate during oxidative stress events and controls the browning of tissues.

Another enzyme in plants, that sometimes of important role under stress, is the oxidation of phenolic compounds, according to its function of other names. This paper may help because the matter of its use in avoiding or miniaturizing brown tissue because of controlling polyphenol oxidase activity, the reduction in polyphenol oxidase, this back to avoiding tissues browning and helping in uptake of nutrients, as shown in a similar study to our results in this point, which showed the decrease in pepper plant PPO level because of SAH;^49^.

H_2_O_2_ is an indirect result because of the effect of super absorber hydrogel on avoiding it possibly avoiding H_2_O_2_ stress-induced elevation because of drought and consequently leads to the amelioration of the physiological health of the plants. Because, as the results show, where^50^, state that it may help with decreased high accumulation including H_2_O_2_ content because of water stress, whereas MDA is one of the plant lipid peroxidation processes, and it has been known as a marker for plants under oxidative stress.

The lipid peroxidation happens when cellular membranes are attacked by reactive oxygen species (ROS) that generate MDA^51^; the reduced content in MDA could be credited to the declining state of oxidative stress in the plant; a decrease in the levels of oxidative stress might result in a decreased lipid peroxidation in the exact line^52^ showed that SAH diminished H_2_O_2_ and MDA content in soybean.

## Conclusion

The research showed that these superabsorbent Na-CMC/PAAm hydrogels were successfully synthesized by gamma radiation-initiated copolymerization and further modified to improve swelling and water retention properties. The amount of Na-CMC to AAm employed and the irradiation dosage were the two most important factors determining the properties of these hydrogels. A perfect ratio of 60/40 resulted in maximum equilibrium swelling values of about 500 g/g with a well-balanced hydrophilic-hydrophobic network structure. Higher content levels of AAm encouraged crosslinking which could enhance gel fraction up to 85% while reducing their capacity for absorbing water because they are non-polar compounds. However, high radiation doses resulted in an increased gel fraction through crosslinking but restricted swelling capacity as well.

Chemical modification by KOH treatment was highly influential in boosting the swelling ratio up to 415 after the second cycle, owing to introducing ionic carboxylate groups that enhanced hydrophilicity and electrostatic repulsions in the network. SEM analysis showed that the changed hydrogels had bigger, pore cavities as opposed to plain and superficial pores in unadjusted ones. These adjusted hydrogels had a more expanded porous architecture with enhanced hydrophilicity for better water retention performance. This indicated that they could hold up to 75% of the absorbed water even after 16 days compared to non-modified hydrogel which lost 50% of its water within only five days.

Optimized composition synergistically worked with KOH modification and controlled radiation cross-linking to result in Na-CMC/PAAm hydrogels which swelled selectively, and possessed high gel content while remaining best for long-term water retention. Therefore, such properties make modified hydrogels as potential alternatives for drought mitigation in agriculture and enhancement of crop productivity in dry regions with limited availability of water. To ascertain the full potentiality of these polymers in sustainable agriculture, it is suggested that further studies should be done on their application in natural soil environments and plant growth.

Under very dry field conditions, the shoot length increase was 18%, root length was increased by 32%, shoot fresh weight rose by 15% and shoot dry weight was raised by 15%. The protein content went up by 19% resulting in a rise of leaf chlorophyll levels to a maximum of 12 per cent; carbohydrate production was enhanced by 13%, yielding an increment of yield. These results showed that the stress enzymes decreased which indicated the suppression of oxidative damage.

## Supplementary Information

Supplementary Information.

## Data Availability

All data generated or analyzed during this study available from the corresponding author on request.
