# Supplementary material for: Radiation-assisted tailoring of swelling behavior and water retention of Na-CMC/PAAm hydrogels for enhancing Beta Vulgaris under drought stress
Source: Sci Rep. 2025 Jan 11;15:1661. doi: 10.1038/s41598-024-83832-3 (PMC11724103; doi:10.1038/s41598-024-83832-3)
Supplement: Supplementary file 1 — Supplementary Information. [file 41598_2024_83832_MOESM1_ESM.docx]

## Supplementary file

**Radiation** **Assisted Tailoring of Swelling Behavior and Water Retention of Na-CMC/PAAm Hydrogels for Enhancing *Beta vulgaris* subsp. *vulgaris* under Drought stress**

Mahmoud A. El-diehy^1^, Ibrahim I. Farghal^1^, Mohamed A. Amin^1^, Mohamed mohamady Ghobashy^2^, Abdelatti I. Nowwar^1^, H. M. Gayed^2^

^1^Botany and Microbiology Department, Faculty of Science, Al-Azhar University, Cairo, Egypt

^2^Radiation Research of Polymer Chemistry Department, National Center for Radiation Research and Technology (NCRRT), Egyptian Atomic Energy Authority (EAEA), Cairo, Egypt.

1. **Experimental**
2. **Pigment and Carotenoid Identification**

The method used for the quantitative determination of chlorophyll was that of **(Vernon and Selly, 1966)** . In such method, one gram aliquot of fresh leaves was cut into small pieces. The pigments were extracted by grinding the cut tissues with suitable amount of glass powder in a mortar using 100 ml of 80% aqueous acetone (v/v). The homogenate crude of plant extract was transferred quantitatively to a Buchner filter with Whatman No.1 filter paper. The filtrate was transferred quantitatively to 100 ml volumetric flask and made up to a total volume of 100 ml using 80% acetone.

The optical density of the plant extract was measured using spectrophotometer of two wave lengths (649 and 665 nm). These are positions in the spectrum where maximum absorption by chlorophyll (a) and (b) occurs. The concentrations of chlorophyll (a), (b) and total chlorophyll in plant extracts were calculated using the equations mentioned by**(Vernon and Selly, 1966)**.

mg chlorophyll (a) / g tissue = 11.63(A665) – 2.39(A649).

mg chlorophyll (b) / g tissue = 20.11(A649) – 5.18(A665).

mg chlorophyll (a + b) / g tissue = 6.45 (A665) +17.72(A649).

For carotenoids, the concentration was carried according to **(Lichtenthaler et al., 1981)** equation:

Car x+c = 1000 × OD470- 1.82 Ca – 85.02 Cb/ 198= mg/g fresh weight.

"A" denotes the reading of optical density.

1. **mation of total phenolic compoundsEsti**

Extraction of phenolic compounds were carried out according to that method was described by **(Diaz and Martin, 1972)** as follows: 1 g of dry defeated ground leaves was extracted in 5-10 ml 80% ethanol for at least 24 hours at 0°C. the alcohol was clarified, the remained residue was re-extracted with 5-10 ml 80% ethanol 3 times. At the end, the clarified extract was completed to 50 ml using 80% ethanol. The colorimetric method of Folin-Denis as described by **(Diaz and Martin, 1972)**was employed for the chemical determination of phenolic compounds as follows:

**Folin-Denis reagent:**

This reagent was prepared by mixing sodium tungstate (100 g) with sodium molybdate (25 g), water (700 ml.), phosphoric acid (50 ml. 85%) and concentrated hydrochloric acid (100 ml) in 1.5 L. conical flask. The flask was attached to a reflux condenser and gently boiled for 10 hours. After cooling at room temperature, lithium sulphate (150 g). water (50 ml.) and a few drops of liquid bromine were added. The mixture was then boiled without condenser for about 15 min. to remove the excess of bromine, then cooled, and diluted to 1 liter and filtered.

**Sodium carbonate saturated solution:**

Sodium carbonate (35%) was prepared at 70-80°C. The solution then cooled, kept overnight and filtered.

**Procedure:**

An aliquot of 0.5 ml of the previous extract and 0.5 ml of Folin-Denis reagent were well mixed in a dry test tube, the tube was thoroughly shaken for 3 min. 1.0 ml of saturated Na_2_CO_3_ solution was added, mixed well and 3 ml of distilled water were added. After one hour, the developed colour was reading at 725 nm using 0.5 ml 80% ethanol and reagents only as a blank.

| Total phenols (mg/100g) = | | | $\text{(X) ppm x Extract volume x 100}$ |
| --- | --- | --- | --- |
|  |  |  | $\text{Sample dry weight x 1000 x 1000}$ |
| X= | Y – 0.01072 |  |  |
|  | 0.00904 |  |  |

Where, y = Optical density.

1. **Determining Free Proline Levels in Plant Tissues:**

The plant content of unfastened proline can be envisioned by the technique of **(Bates et al., 1973)**. In this technique, 0.5 gram of dry tissues become grounded in 10 ml (3%) sulfosalicylic acid. The extract become filtered with Whatman No.2 filter paper. A aggregate of two ml of filtrate, 2 ml acid ninhydrin (It can be prepared through heating 1.25 gm ninhydrin in 30 ml glacial acetic acid and 20 ml 6M phosphoric acid, with shaking, till melted, then cooled) and a couple of ml of glacial acetic acid in a check tube have been boiled inside the water tub for 1 hour, then the response become stopped in an ice tub. Then, the tube content become extracted with 4 ml toluene, and mixed strongly by using a test tube stirrer for 15-20 sec. The colored upper layer extracted with toluene became separated from the aqueous solution, warmed to room temperature and the diploma of colour absorption at a wavelength of 520 nm by means of UV- colorimeter. Toluene become used as a clean and proline for the coaching of the usual curve, the proline concentration become calculated from the standard curve based totally on the dry weight of the pattern as follows:

| Mg/g proline = | (X) PPM * ml Extract volume | |
| --- | --- | --- |
|  | 2 * Sample dry weight * 100 |  |

1. **Extraction and Quantification of Catalase, Peroxidase, and Polyphenol Oxidase Enzymes**

**Extraction:**

The plant materials used for estimation of catalases (CAT) and peroxidases (POX) enzymes were the terminal buds in addition to the first and second young leaves. In this regard, 2 g of the plant materials were homogenized with 10 ml of phosphate buffer pH 6.8 (0.1 M), then centrifuge at 2 ^o^C for 20 min at 20000 rpm in a refrigerated centrifuge. The clear supernatant (containing the enzymes) was taken as the crude enzymes source **(MUKHERJEE, 1983).**

- **Determination of Catalase Activity**

Catalases activity was assayed according to that method of **(Chen et al., 2000).** The reaction mixture with final volume of 10 ml containing 40 µl crude enzyme extract was added to 9.96 ml H_2_O_2_ phosphate buffer pH 7.0 (0.16 ml of 30% H_2_O_2_ to 100 ml of 50 mM phosphate buffer). Catalases activity was determined by measuring the rate change of H_2_O_2_ absorbance in 60 second with a UV- spectrophotometer (Jenway) at 250 nm. The blank sample was made by using buffer instead of enzyme extract. One unit of enzyme activity was defined as the amount of the enzyme that reduced 50% of the H_2_O_2_ in 60 second at 25 ^0^C **(Kong et al., 1999).**

- **Peroxidase (POX) activities:**

Peroxidases activity were assayed using the solution that containing 5.8 ml of 50 mM phosphate buffer pH 7.0, 0.2 ml of the enzyme extract and 2 ml of 20 mM H_2_O_2_ after addition of 2 ml of 20 mM pyrogallol, the rate of increase in absorbance as pyrogallol was determined spectrophotometrically by UV- spectrophotometer (Jenway) within 60 second at 470 nm and 25 ^0^C **(Pütter, 1974).** One unit of enzyme activity was defined as the amount of the enzyme that catalyzed the conversion of one micromole of H_2_O_2_ per minute at 25 ^0^C **(Kong et al., 1999)**. The blank sample was made by using buffer instead of enzyme extract.

In case of enzyme assay, volume at zero time was taken as blank and the activity of the enzyme / g fresh weight / hour was expressed as (∆ × T v × 60 min) / (t × v × F.Wt.) where, ∆ is the absorbance of the sample after incubation minus the absorbance at zero time, while (Tv) is the total volume of filtrate, (t) is the time (minutes) of incubation with substrate and (v) is the total volume of filtrate taken for incubation and (F.Wt.) is the fresh weight used **(Fick and Qualset, 1975).**

- **Polyphenol oxidase (PPO) activity:**

The activities of polyphenoloxidase enzyme were determined according to the method adopted by **(Kar and Mishra, 1976).**The reaction mixture contained 1.0 ml of crude enzyme extract, 1.0 ml of 0.2 M sodium phosphate buffer at pH 7.0, 10 ml of 0.001 M catechol (C_6_H_4_(OH)_2_) and 3.0 ml distilled water.

The absorbance was measured at 495 nm by (Unico 2000) and the optical densities were recorded within 60 seconds. Polyphenoloxidase activities were expressed as changes in the optical density/min./g fresh weight. In each determination, control treatment (blank) contained all chemical reagents except the enzyme extract, its recorded value was subscribed from all readings.

In case of enzyme assay, volume at zero time was taken as blank and the activity of the enzyme / g fresh weight / hour was expressed as (∆ x T v x 60 min) / (t x v x F.Wt.) where, (∆) is the absorbance of the sample after incubation minus the absorbance at zero time, (Tv) is the total volume of filtrate, (t) is the time (minutes) of incubation with substrate, while (v) is the total volume of filtrate taken for incubation and (F.Wt.) is the fresh weight used **(Fick and Qualset, 1975).**

1. **Determination of Soluble Carbohydrates:**

Contents of soluble carbohydrates were determined using anthrone technique according to **(Umbreit, 1957)**.Ten ml of the filtrate of each sample were mixed with 2 gm of activated charcoal and shaken well for 15 minutes followed by filtration used Whatman No.1 filter paper. The clear filtrate was diluted quantitatively by distilled water. Two ml of diluted filtrate were transferred to test tube and add 4 ml of freshly prepared anthrone reagent (2 g anthrone/1 of 95% pure sulphuric acid). The tube was then placed in a boiling water bath for 3 minutes. After which it was left to cool. The developed color was measured using spectrophotometer at 620 nm. A blank mixture containing distilled water and reagent of anthrone was used to setup the apparatus at zero O.D.

1. **Quantification of Water-Soluble Proteins**

Based on the work by **(Lowry et al., 1951)**, casein was used as the standard protein for this method.

Solution A: This is a 2% answer of sodium carbonate (Na2CO3) in zero.1 N sodium hydroxide (NaOH).

Solution B: This is a solution made through dissolving zero.5 grams of copper sulfate (CuSO4) in a 1% answer of sodium potassium tartrate Solution C: This solution is made by using mixing 50 ml of solution A with 1 ml of solution B. This mixture ought to be organized right earlier than measuring the protein Solution D: This solution is made by using diluting the folin reagent (from BDH) with distilled water in a 1:three extent ratio.

Here's how to do the test:

In a take a look at tube, mix 1 ml of the plant extract you need to test for protein with 5 ml of solution C. Stir the combination and permit it cooled for ten mins. Then fast add Five ml of D, blend it in, and permit it sit down for some other 30 minutes. After that, measure the colour intensity the use of a tool that reads light at a wavelength of 750 nm.

**Reference**

AEBI, H. 1984. [13] Catalase in vitro. Methods in enzymology. Elsevier.

BATES, L., WALDREN, R. A. & TEARE, I. 1973. Rapid determination of free proline for water-stress studies. Plant and soil, 39, 205-207.

CASTILLO, F. J., PENEL, C. & GREPPIN, H. 1984. Peroxidase release induced by ozone in Sedum album leaves: involvement of Ca2+. Plant physiology, 74, 846-851.

KONG, F., HU, W., CHAO, S., SANG, W. & WANG, L. 1999. Physiological responses of the lichen Xanthoparmelia mexicana to oxidative stress of SO2. Environmental and Experimental Botany, 42, 201-209.

LICHTENTHALER, H., BUSCHMANN, C., DÖLL, M., FIETZ, H.-J., BACH, T., KOZEL, U., MEIER, D. & RAHMSDORF, U. 1981. Photosynthetic activity, chloroplast ultrastructure, and leaf characteristics of high-light and low-light plants and of sun and shade leaves. Photosynthesis research, 2, 115-141.

LOWRY, O. H., ROSEBROUGH, N. J., FARR, A. L. & RANDALL, R. J. 1951. Protein measurement with the Folin phenol reagent. Journal of biological chemistry, 193, 265-275.

MATTA, A. & DIMOND, A. 1963. Symptoms of Fusarium wilt in relation to quantity of fungus and enzyme activity in tomato stems. Phytopathology, 53, 574-&.

SAID, A., NAGUIB, M. & RAMZY, M. 1964. Sucrose determination as a means of estimation of the draw back tax on exported Halawa Tehinia. Bull. Fac. Sci. Cairo Univ, 39, 207-216.

SINGLETON, V. L., ORTHOFER, R. & LAMUELA-RAVENTÓS, R. M. 1999. [14] Analysis of total phenols and other oxidation substrates and antioxidants by means of folin-ciocalteu reagent. Methods in enzymology. Elsevier.

SNEDECOR, G. W. & COCHRAN, W. G. 1989. Statistical methods, 8thEdn. Ames: Iowa State Univ. Press Iowa, 54, 71-82.

UMBREIT, W. 1957. Manometric techniques.

VERNON, L. P. & SELLY, G. R. 1966. The chlorophylls. Acad. Press, New York, London.
